# Catalyst Influence on the Crystallization Behaviors and Mechanical Properties of Polyethylene Vitrimers

**DOI:** 10.1021/acs.macromol.5c02354

**Published:** 2025-10-27

**Authors:** Sara Valdez, Zhe Qiang

**Affiliations:** School of Polymer Science and Engineering, The University of Southern Mississippi, Hattiesburg, Mississippi 39406, United States

## Abstract

Polyethylene (PE)-derived vitrimers represent a promising class of reprocessable materials that combine the desirable properties of semicrystalline polyolefins with dynamic covalent networks. In this study, we investigate the influence of transesterification catalyst identity and loading on the crystallization behavior and mechanical properties of vitrimers formed from maleated linear low-density PE crosslinked with diglycidyl ether bisphenol A (DGEBA). Four catalysts, including zinc­(II) stearate, zinc­(II) acetylacetonate, zinc­(II) acetate, and manganese­(II) acetate, were studied across a range of loadings. Through thermal and mechanical characterization, we demonstrate that catalyst selection can play a critical role in controlling network formation and crystallization kinetics, in part due to their ability to act as nucleating agents and influence crystallization activation energies across varying cooling rates. These insights contribute to a deeper understanding of how dynamic chemistry of vitrimers can be leveraged to impact processing conditions and mechanical performance of semicrystalline polyolefin systems.

## Graphical abstract

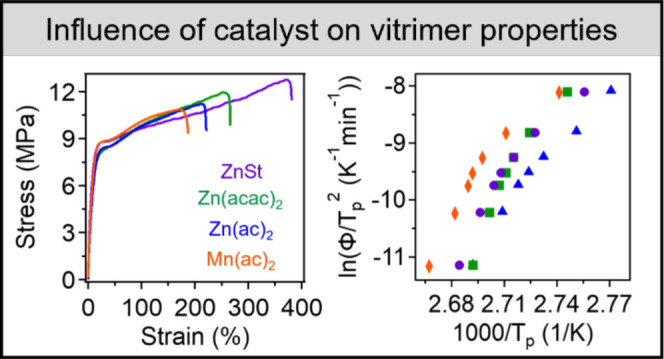

## Introduction

Polyolefins (POs) are widely used in various applications such as packaging, textiles, and automotive components, making them a common part of everyday life.^1,2^ Among them, polyethylene (PE) and polypropylene (PP) lead the global market due to their low production costs, reliable mechanical performance, and excellent chemical resistance, qualities largely stemming from their semicrystalline nature.^3,4^ Controlling the crystallinity of POs is a key area of research, as it directly influences their mechanical, thermal, and optical properties. Extensive studies have shown that both polymer structure and processing conditions significantly affect crystallization behavior and kinetics.^3,5^ In industrial settings, rapid cooling combined with extensional or shear flows is often used to tune the crystalline morphology of polyolefins, allowing for control over their macroscopic properties and performance.^5–9^ As a result, understanding how POs crystallize under non-isothermal conditions is crucial for designing efficient manufacturing processes and tailoring materials to meet specific application requirements.

While plastics play a vital role in modern life, their improper end-of-life management causes significant environmental and health challenges. In response, there have been growing efforts in recent years to improve plastic sustainability. Particularly, a promising route is associated with the emergence of dynamic polymer networks, which leverage reversible crosslinking chemistries to enable material reprocessability and recyclability.^10–15^ These materials are more specifically known as covalent adaptable networks (CANs), and they are categorized based on their bond exchange mechanisms as either “dissociative” or “associative” networks. When CANs retain their crosslink density during reprocessing conditions via an associative bond exchange mechanism, they can be classified as vitrimers. Although crosslinked polyethylene (PEX) exists and is used in some applications, the development of PE-based vitrimers is part of a broader effort in polymer science to improve the sustainability of commodity plastics. By incorporating dynamic covalent cross-links, PO-derived CANs offer potential pathways for reprocessing and upcycling, aligning with current efforts in the field toward more circular materials.

PO-derived vitrimers involve a wide range of dynamic chemistries including transesterification,^16–19^ disulfide exchange,^20–27^ boronic ester transesterification/dioxaborolane metathesis,^28–32^ and others.^33–38^ Much of the literature has focused on developing vitrimer chemistries and investigating how dynamic crosslinks, catalysts, and reprocessing conditions influence bulk properties such as thermal stability, mechanical performance, and flow behavior. In parallel, significant efforts have been made to understand network rearrangement and bond exchange dynamics, particularly in relation to thermal transitions (e.g., topological freezing temperature),^23,33,35,39^ stress relaxation,^40–45^ and creep resistance,^22,26,30,46–48^ guiding the design of vitrimer networks. Despite these advances, there remains limited understanding of how crystallinity of PO-derived vitrimers evolves during processing conditions, especially in relation to the extent of crosslinking and catalyst presence. This gap is particularly important where crystallization and network formation can influence each other in complex and sometimes even competing ways. Studies have shown that the degree of crystallinity, as well as the size of spherulites and lamellae, strongly influences a material’s thermal and mechanical properties, while crosslinking can restrict polymer chain mobility, often leading to a reduction in overall crystallinity. Moreover, a recent work found that zinc stearate (ZnSt) and 1,5,7-triazabicyclo[4.4.0]­dec-5-ene (TBD) could influence PO vitrimer crystallization by acting as a nucleating agent.^49^ These results suggest that a further understanding of how dynamic crosslinking chemistry influences crystallization behavior is essential to advance the design and application of PO-derived vitrimers.

In this study, linear low-density polyethylene (LLDPE) grafted with maleic anhydride is dynamically crosslinked with diglycidyl ether of bisphenol A (DGEBA) via esterification chemistry. To investigate the catalyst effects on the cross-linking process, four metal-based catalysts are evaluated: zinc­(II) stearate, zinc­(II) acetylacetonate, zinc­(II) acetate, and manganese­(II) acetate with varied loading content between 0.2 and 3 wt %. The Jeziorny-modified Avrami model, along with the Kissinger method, was used to understand the nonisothermal crystallization behaviors of the dynamic networks. It was found that both catalyst identity and loading can impact the resulting gel content, degree of crystallinity, and lamellar thickness, which in turn influence their mechanical properties. The identity of the catalysts directly dictated the crystallization activation energies, leading to distinct nucleation- and growth-dominated regimes depending on the applied cooling rate. Overall, this study offers important insights that advance the design of semicrystalline PO-derived dynamic networks, by elucidating how catalyst selection and loading can govern crystallization behaviors and network development.

## Experimental Section

### Materials

TYMAX maleated LLDPE, grade GT4300 (LLDPE), containing approximately 1.5 wt % MA (∼0.43% of monomers contain MA), confirmed by titration in a previous study,^50^ was obtained from Westlake Chemical. Bisphenol A diglycidyl ether (DGEBA), zinc­(II) stearate (ZnSt), manganese­(II) acetate (Mn­(ac)_2_), zinc­(II) acetate (Zn­(ac)_2_), and zinc­(II) acetylacetonate (Zn­(acac)_2_) were purchased from Sigma-Aldrich. ACS Reagent grade xylenes was purchased from Fisher Chemical. Chemicals were used as received.

### Sample Preparation

All samples were compounded using an Xplore MC5 microcompounder. Specifically, vitrimer samples were compounded at 200 °C with a screw speed of 20 rpm and a recirculation time of 10 min. The formulations of vitrimers consisted of LLDPE (2.5 g) and DGEBA (65 mg, 1:1 ratio of epoxy/MA), and the catalyst. Formulations with ZnSt and Zn­(acac)_2_ were made with four catalyst loadings including 0.2 wt % (5 mg), 0.5 wt % (12.5 mg), 1 wt % (25 mg), and 2 wt % (75 mg), with respect to LLDPE. Formulations with Zn­(ac)_2_ and Mn­(ac)_2_ were made with one catalyst loading, 1 wt % (25 mg). To create vitrimers consisting of only the cross-linked fraction, the soluble (uncross-linked) fraction was removed via solvent extraction with xylenes at 120 °C. The full vitrimer sample was completely submerged in the hot xylenes for 24 h. Subsequently, the xylenes (containing the dissolved soluble fraction) was removed by pipetting, yielding LLDPE_V_-insol. To add the catalyst back into the insoluble fraction, LLDPE_V_-insol was compounded again (with the same conditions outlined above) and 1 wt % ZnSt was added to yield LLDPE_V_-insol-ZnSt 1 wt %. All components were added to the compound at the same time. In addition to vitrimer samples, physical blends of LLDPE and the catalyst were compounded at 200 °C with a screw speed of 50 rpm and a recirculation time of 6 min. Similar to the vitrimers, formulations made with ZnSt and Zn­(acac)_2_ were made with four catalyst loadings including 0.2 wt % (5.4 mg), 0.5 wt % (13.5 mg), 1 wt % (27 mg), and 3 wt % (81 mg), whereas blends made with Zn­(ac)_2_ and Mn­(ac)_2_ were prepared with 1 wt % (27 mg) catalyst.

Sample bars for dynamic mechanical analysis (DMA), tensile testing, and Fourier transform infrared (FTIR) spectroscopy were melt pressed with a Carver 4386 press. The bars were made in a mold (2 × 0.5 × 0.05 cm) sandwiched between polytetrafluoroethylene (PTFE) release films. All samples were pressed at 190 °C. The samples were heated without pressure for ∼10 min to get to temperature and were then pressed for 5 min, the first 3 min under 3 ton of pressure and the final 2 min at 6 ton. Samples were cooled on an aluminum bench with a steel heat sink that continued compressing the mold for ∼12 min before the bars were removed from the mold. We note throughout this work, the naming scheme for samples is “LLDPE (subscript V or B depending on if the sample is a vitrimer or physical blend, respectively)-catalyst type catalyst loading.”

### Characterization

FTIR spectroscopy was performed by using a PerkinElmer Frontier spectrometer equipped with an attenuated total reflectance (ATR) accessory. Each sample was analyzed by averaging 32 scans at a spectral resolution of 4 cm^–1^, with the range from 4000 to 600 cm^–1^. To ensure consistency across samples, spectra were normalized to the characteristic LLDPE band at 2916 cm^–1^, corresponding to the asymmetric C–H stretching vibrations. DMA was conducted using a TA Instruments Discovery DMA 850, with data collected and processed with TRIOS software. All measurements employed a film tension clamp. Oscillatory temperature ramp experiments were performed under a constant strain of 0.05% at a frequency of 1.0 Hz, with a preload force of 0.01 N. The temperature range measured was 25–270 °C at a ramp rate of 3 °C/min.

Uniaxial tensile tests on the LLDPE vitrimer materials were conducted by using a Mark-10 F105-EM test frame equipped with a Series FS05-50 force sensor (250 N capacity), operated at a constant strain rate of 10 mm/min. Data analysis was performed using Igor Pro 9.02, where Young’s modulus was determined from the slope of the linear region preceding yield, the ultimate tensile strength (UTS) was the highest stress achieved, strain at break was reported as the strain (%) that the sample fractured, and toughness was calculated by integrating the area under the stress–strain curve for each sample. Differential scanning calorimetry (DSC) measurements were carried out with a TA Instruments Discovery DSC 250. Approximately 8–10 mg of extrudate from the microcompounder was used for each sample. Data acquisition and analysis were performed by using TRIOS software. For nonisothermal crystallization kinetics, samples were first heated to 200 °C at a rate of 10 °C/min and held isothermally for 5 min to eliminate thermal history. Subsequently, the samples were cooled to 25 °C at various cooling rates (Φ = 2, 5, 8, 10, 13, 20, and 40 °C/min), with a 5 min isothermal hold at 200 °C between each cooling step.

Gel fractions were calculated using the following equation

1 $$gel\;fraction=\frac{w_{f}}{w_{i}}\times 100\%$$

where *w*_i_ and *w*_f_ are the masses before and after solvent extraction, respectively.

The degree of crystallinity (χ_c_) for all samples was calculated from the second heating cycle, following cooling at 2 °C/min, using the equation below

2 $$\chi_{c}=\left(\frac{\Delta H_{f}}{\Delta H_{f}^{o}}\right)\times 100\%$$

where Δ*H*_f_ is the enthalpy of fusion and Δ*H*_f_^o^ is the standard enthalpy of fusion for PE which is equal to 293 J g^–1^.

Crystal lamellar thickness (*L*) and the corresponding *L* distributions were determined using the same second heat cycle, as previously mentioned. Equation 3 is the Gibbs–Thomson equation which was used to calculate *L* and eq 4 describes lamellar thickness distributions.

3 $$T_{m}=T_{m}^{o}\left(1-\frac{2\sigma_{e}}{\Delta H_{f,c}^{o}L}\right)\%$$

4 $$\frac{1}{M}\frac{dM}{dL}=\frac{dE}{dT}\frac{{\left(T_{m}^{o}-T_{m}\right)}^{2}\rho_{c}}{2\sigma_{c}T_{m}}\%$$

here, *T*_m_ represents the peak melting temperature (in Kelvin) corresponding to a crystalline lamella of thickness *L*. *T*_m_^o^ is the equilibrium melting temperature of PE (415 K), and σ_e_ denotes the surface energy of the basal plane of a lamellar crystal (6.09 × 10^–6^ J·cm^–2^). The term Δ*H*_f,c_° refers to the enthalpy of fusion in the crystalline phase. *M* is the mass of the crystalline portion in the sample, d*E*/d*T* is the energy required to melt the incremental mass d*M* of the crystalline phase, and ρ_c_ is the density of the crystalline phase, approximately 1 g·cm^–3^.

Crystallization kinetics were investigated by using the Jeziorny-modified Avrami model. The Avrami model describes material transformation from one phase to another with a constant temperature and has been widely utilized to investigate crystallization of semicrystalline polymers and can be extended to nonisothermal conditions.^7,51–57^ The Avrami equation can be found below (eq 5) and a rearranged form (eq 6) was used for creating the Avrami plot.

5 $$X\left(t\right)=1-\exp\left(-Kt^{n}\right)$$

6 log[−ln(1−X(t))]=log⁡K+nlog⁡t

In these equations, *n* is the Avrami exponent, which reflects the nucleation mechanism and the geometry of crystal growth, and *K* is the Avrami constant, representing the overall crystallization rate. *X*(*t*) represents the instantaneous relative crystallinity at time t and is calculated using eq 7.

7 $$X\left(t\right)=\frac{Q_{t}}{Q_{\infty }}=\frac{\int_{0}^{t}\left(\frac{dh}{dt}\right)dt}{\int_{0}^{\infty }\left(\frac{dH}{dt}\right)dt}$$

In this context, *Q*_*t*_ is the instantaneous heat flow, *Q*_∞_ represents the total heat released during the crystallization process, and d*H*/d*t* is the rate of the enthalpy change at a given moment. Under nonisothermal conditions, the crystallization time (*t*) can be determined from the crystallization temperature (*T*) using the following equation

8 $$t=\frac{T_{onset}-T}{\phi }$$

where, *T*_onset_ represents the crystallization onset temperature and ϕ is the cooling rate. Crystallization half-time (*t*_1/2_) is the amount of time it takes for the relative crystallization to equal 50% and can be determined by using the development of crystallinity plots and then converting the temperatures to crystallization time, *t*, using eq 8. *t*_1/2_ values for all samples can be found in the Supporting Information. Because nonisothermal conditions were used in this study, it has been established that the Avrami constant should be corrected with the cooling rate to give the Jeziorny-modified Avrami constant (*K*′) which can be determined using eq 9.^55,58,59^

9 $$\log K^{\prime }=\frac{\log K}{\phi }$$

Crystallization activation energies were determined using the Kissinger equation shown below^60^

10 $$\ln\left(\frac{\phi }{T_{p}^{2}}\right)=C-\frac{\Delta E}{RT_{p}}$$

where *C* is a constant, *R* is the gas constant (8.314 J/mol), *T*_p_ is the peak crystallization temperature in K, and Δ*E* is the activation energy.

## Results and Discussion

Figure 1a shows the chemical structures of the maleated LLDPE and crosslinker, DGEBA, used to create the dynamic networks for this study. The crosslinking step is achieved through a ring-opening reaction of the maleic anhydride (MA) with the epoxy groups of DGEBA. Catalyst presence allows for the ester bonds to be reversible with elevated temperatures via Lewis acid activation and is illustrated in Figure 1b. Namely, the transition metal salts coordinate with the carbonyls, activating them for nucleophilic attack from any free hydroxyls in the system. The mechanism of this reaction allows the formation of vitrimers, which can exhibit flowability at elevated temperatures while retaining cross-link density. Within this work, four different catalysts were studied including zinc­(II) stearate (ZnSt), zinc­(II) acetylacetonate (Zn­(acac)_2_), zinc­(II) acetate (Zn­(ac)_2_), and manganese­(II) acetate (Mn­(ac)_2_) and their chemical structures can be found in Figure 1c. The zinc-based catalysts were selected as they are most commonly used in catalyzing esterification reactions, particularly in the context of vitrimers.^17,19,61,62^ While Mn­(ac)_2_ is a less common catalyst in the vitrimer field, it still exhibits proven catalytic activity, thermal stability, and generally lower toxicity (particularly with aquatic life) over its zinc counterparts.^63^ For this work, all networks were prepared in the same way by using reactive extrusion with an epoxy/MA molar ratio of 1:1. ZnSt and Zn­(acac)_2_, which were investigated with catalyst loadings of 0.2, 0.5, 1, and 3 wt % (with respect to the mass of LLDPE), while Zn­(ac)_2_ and Mn­(ac)_2_ were only formulated with catalyst loadings of 1 wt %.

**Figure 1:**
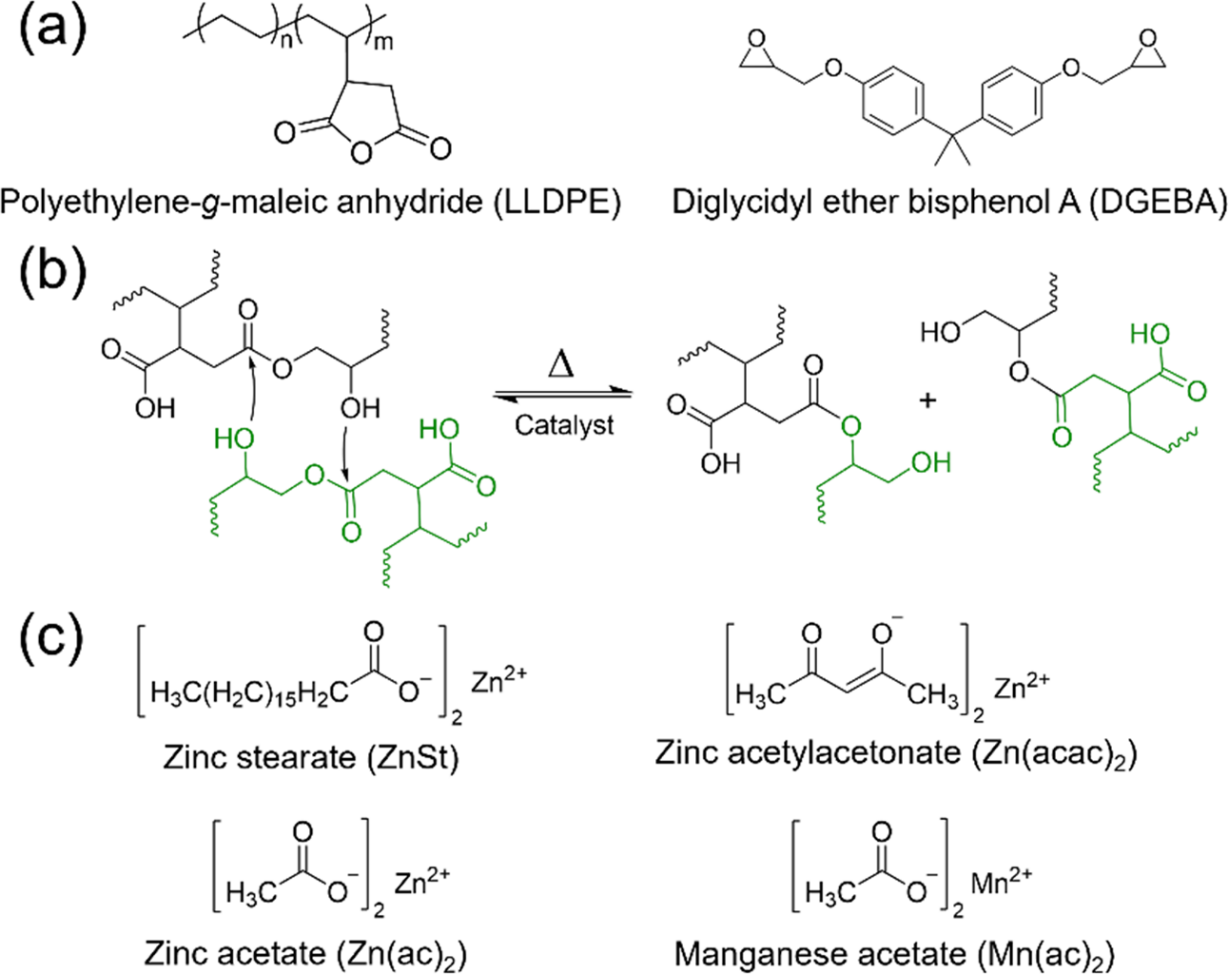
(a) Chemical structures of the components of the dynamic network: LLDPE and DGEBA (crosslinker) and (b) illustration of the dynamic chemistry, transesterification. (c) Chemical structures of the catalysts.

FTIR spectroscopy was used to confirm the crosslinking reaction, and the spectra for vitrimers made with ZnSt (LLDPE_V_-ZnSt) and Zn­(acac)_2_ (LLDPE_V_-Zn­(acac)_2_) can be found in Figure S1 along with the spectrum of neat LLDPE. Within these spectra, characteristic bands for PE can be seen at 2916 (asymmetric C–H stretching), 2850 (symmetric C–H stretching), 1473 (CH_2_ scissoring), and 719 cm^–1^ (CH_2_ rocking). To understand the esterification reaction, a zoomed in spectrum spanning 1500–1800 cm^–1^, is shown in Figure S1b,d. Here, the neat LLDPE band at 1790 cm^–1^ is due to C=O stretching of the ring-closed MA (grafted on LLDPE) and the band at 1712 cm^–1^ corresponds to C=O stretching from carboxylic acids, resulting from ring-opened, unreacted MA (grafted on LLDPE). The reduction/disappearance of these peaks in the vitrimer spectra, in addition to the growth of the band at 1740 cm^–1^ (C=O stretching from esters), indicates the formation of crosslinks. However, it is difficult to quantitatively determine esterification reaction yields with FTIR due to the overlap of the bands at 1740 and 1712 cm^–1^. Additionally, the presence of DGEBA can be seen in the vitrimer samples via the band at 1510 cm^–1^ corresponding to aromatic C–C stretching.

Dynamic mechanical analysis (DMA) was run on the vitrimer samples to further demonstrate network formation. The storage modulus (*E*′) was monitored over elevated temperatures ranging from 25–270 °C (Figure S2). From theis data, it is observed that all ZnSt and Zn­(acac)_2_ vitrimers have a rubbery plateau beginning around 125 °C, indicative of network formation. The specific *E*′ values for the rubbery plateaus can be found in Table S1. The storage modulus plot for neat LLDPE is shown in Figure S2a, where there is no plateau of *E*′ upon crystalline melting and the material continues to flow. Notably, PE_V_-ZnSt *E*′ ranges from 1.12 MPa (0.2 wt %) to 1.27 MPa (1 wt %) compared to the much larger range for PE_V_-Zn­(acac)_2_ of 1.06 MPa (0.2 wt %) to 1.81 MPa (1 wt %). These results suggest that networks made with the presence of ZnSt likely have more comparable crosslink densities despite catalyst loading, whereas those created with Zn­(acac)_2_ have a higher dependence on catalyst loading to achieve increased network crosslink density.

To understand the impact of catalyst type and loading on the mechanical properties of the vitrimers, uniaxial tension tests were performed, and representative stress–strain curves for LLDPE_V_-ZnSt and LLDPE_V_-Zn­(acac)_2_ can be found in Figure 2a. The vitrimer samples and neat LLDPE were run in triplicate, and those results are shown in Figure S3; their Young’s modulus, ultimate tensile strength (UTS), strain at break, and toughness are listed in Table S2. Upon conversion of LLDPE to vitrimers, the networks were able to retain similar UTS values. All vitrimer samples showed reduced Young’s modulus compared to neat LLDPE (147.6 MPa, Figure 2b). For LLDPE_V_-ZnSt, moduli were 90.0, 92.2, 140.5, and 115.3 MPa at catalyst loading content of 0.2, 0.5, 1, and 3 wt %, respectively, while PE_V_-Zn­(acac)_2_ exhibited 99.8, 97.5, 92.6, and 96.6 MPa at the same loadings. Similar decrease in the elastic modulus have been previously reported^38,64^ where this decrease is likely a result of a decrease in the degree of crystallinity (χ_c_) or potentially the results of more complex interplay between crystallinity, gel fraction, and resulting system heterogeneity.

**Figure 2:**
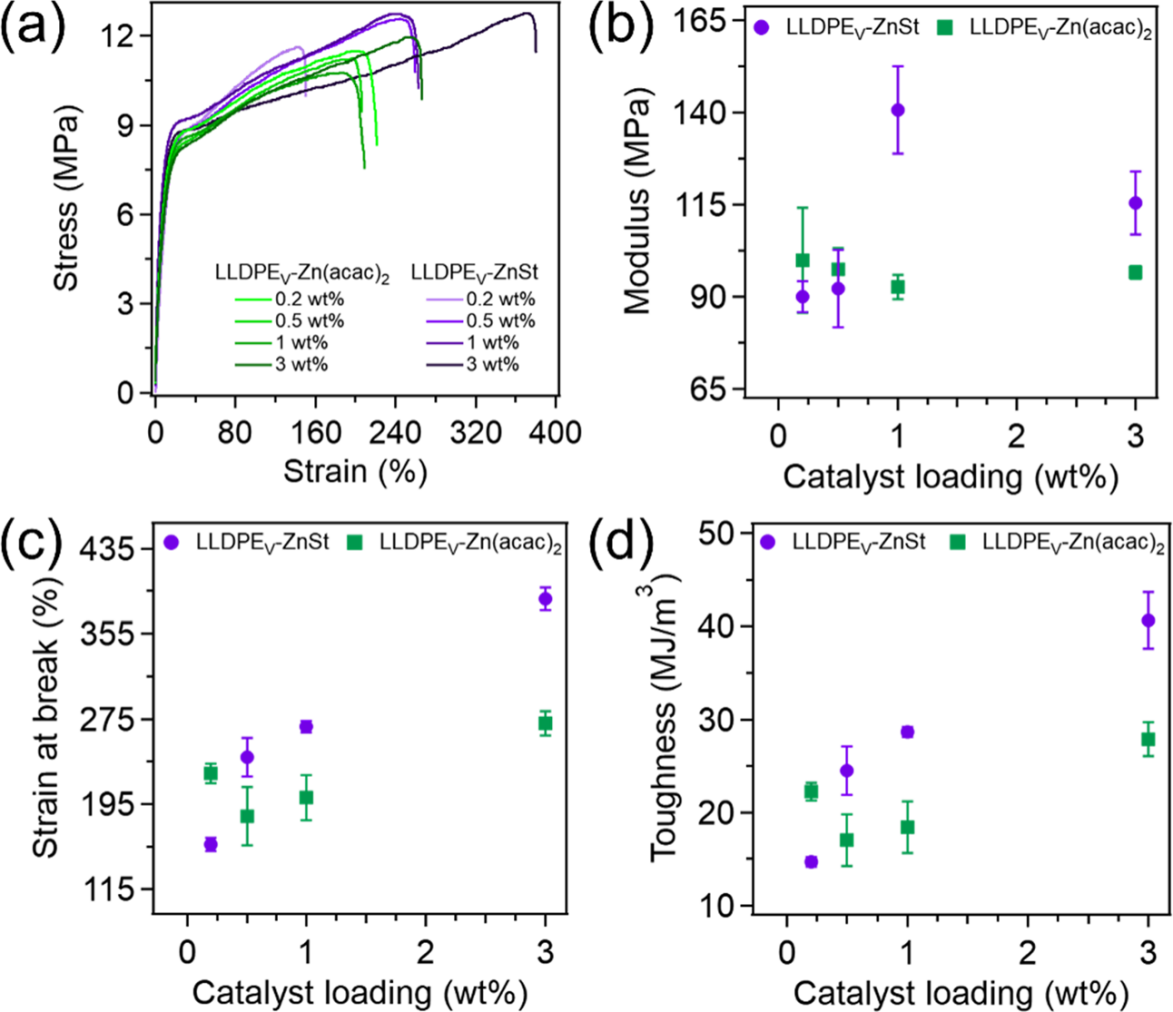
(a) Stress–strain curves, (b) plot of Young’s modulus (c) plot of strain at break, and (d) plot of toughness for LLDPE_V_-ZnSt and LLDPE_V_-Zn­(acac)_2_ with catalyst loadings ranging from 0.2–3 wt %.

All vitrimer samples exhibited reduced strain at break compared with neat LLDPE (724.8%, Figure S3) as shown in Figure 2c. LLDPE_V_-ZnSt fractured at 157%, 239%, 268%, and 388% for 0.2, 0.5, 1, and 3 wt %, respectively, while LLDPE_V_-Zn­(acac)_2_ fractured at 224%, 184%, 201%, and 271% over the same loadings. A similar trend was observed in toughness (Figure 2d), which decreased from 75.5 MJ m^–3^ for neat LLDPE to 14.7–40.8 MJ m^–3^ for LLDPE_V_-ZnSt and 17.0–27.9 MJ m^–3^ for LLDPE_V_-Zn­(acac)_2_ across 0.2–3 wt % loading. The reduction in strain at break is consistent with an earlier onset of strain hardening, attributed to altered stress transmission pathways in the vitrimer network. Crosslinking limits the ability of amorphous chains to dissipate stress, leading to more direct transmission to crystalline domains and network junctions. While reduced chain mobility contributes to stiffness, the strain hardening behavior in this case is more closely linked to stress transmission efficiency.^65,66^ Interestingly, increasing catalyst loading enhances the extensibility of the vitrimer system, which appears counterintuitive given the DMA results suggesting that higher catalyst concentrations can increase cross-link density—typically associated with reduced extensibility. This behavior suggests that catalyst loading may influence the formation of polymer crystalline structures, which in turn governs the stress distribution within the vitrimer network. Generally, the χ_c_ and lamellar thickness (*L*) are interrelated, with thicker lamellae typically associated with increased χ_c_. Studies have shown that larger lamellae contribute to higher stiffness and resistance to deformation, whereas thinner lamellae may enable more deformation mechanisms (e.g., microbuckling or slippage), which can enhance the ductility of the material.^67–70^ This relationship is further complicated by the introduction of dynamic bonding, which can inhibit crystal growth and alter crystallite size, thereby influencing both lamellar morphology and mechanical behavior.^70–74^ To fully understand these effects, it is necessary to investigate how catalyst identity and loading content impact vitrimer crystallization behaviors and the gel content as each contributes to the sample mechanical response.

DSC was used to understand the catalyst and cross-linking impacts on the crystallization and melting behaviors of LLDPE-derived vitrimer samples. Figure 3a,e shows the melting events for vitrimers made with ZnSt and Zn­(acac)_2_, respectively. All of the vitrimer melting endotherms are bimodal, suggesting a mixed component system. From a previous study,^49^ it is known that the higher temperature melting peak around 122 °C can be attributed to the uncross-linked portion of LLDPE, while the lower melting temperature (*T*_m_) is that of the cross-linked fraction (neat LLDPE has *T*_m_ of 122.6 °C). *T*_m_s for all of the samples are listed in Table S3. As the amount of the catalyst is increased, the peak corresponding to the uncrosslinked fraction begins to decrease in all vitrimer endotherms, indicating that more of the LLDPE is being crosslinked to form the vitrimer. For the materials made with ZnSt, it is observed that increasing the catalyst loading has a limited effect on the *T*_m_ and only slightly reduces the *T*_m_ of uncrosslinked LLDPE when 3 wt % of the catalyst is added and the specific temperatures are 0.2 wt %: 112 and 122 °C, 0.5 wt %: 113 and 123 °C, 1 wt %: 112 and 122 °C, and 3 wt %: 111 and 121 °C. However, for networks made with Zn­(acac)_2_, the shift in *T*_m_s is more pronounced with varied loading content, including 0.2 wt %: 112 and 124 °C, 0.5 wt %: 112 and 124 °C, 1 wt %: 111 and 124 °C, and 3 wt %: 110 and 124 °C. A decrease in the *T*_m_ indicates the formation of more imperfect crystalline regions during crystallization, and these defects reduce the energy required to disrupt the crystal lattice, making the material easier to melt. These results indicate that the crosslinked regions in the vitrimers promote the formation of less thermally stable crystallites compared to neat LLDPE, which is consistent with the previous results that crosslinking restricts chain mobility, thereby hindering the development of crystalline structures. For the uncross-linked regions, ZnSt appears to have minimal influence on the crystalline structure, whereas Zn­(acac)_2_ promotes the formation of more thermally stable crystallites compared to neat LLDPE. Plots showing χ_c_ as a function of catalyst loading can be found in Figure 3b,f for ZnSt and Zn­(acac)_2_, respectively (Table S3). All vitrimers show a decrease in χ_c_ from neat LLDPE (39.3%). LLDPE_V_-ZnSt had χ_c_s of 32.8%, 33.8%, 31.6%, and 29.9% for 0.2, 0.5, 1, and 3 wt %, respectively, while LLDPE_V_-Zn­(acac)_2_ had χ_c_s of 29.5%, 34.5%, 30.8%, and 31.5% for the same loadings. Noteworthily, after an initial decrease in χ_c_, due to restricted chain mobility from network formation, increasing ZnSt loading continues to suppress χ_c_, whereas Zn­(acac)_2_ promotes higher crystallinity. Interestingly, both catalysts yield the highest χ_c_ at 0.5 wt % loading, suggesting an optimal balance between crosslink formation and crystalline order.

**Figure 3:**
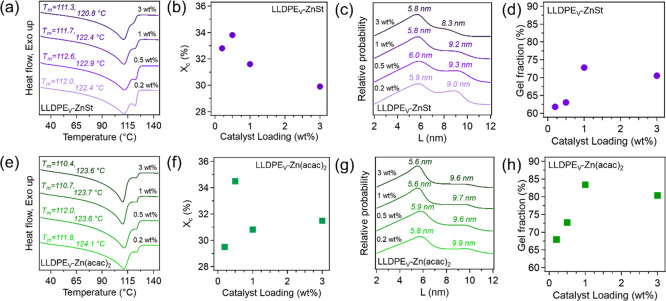
(a,e) DSC thermograms showing melt peaks, (b,f) plot of χ_c_, (c,g) lamellar thickness distributions, and (d,h) plot of gel fraction for LLDPE_V_-ZnSt and LLDPE_V_-Zn­(acac)_2_ with catalyst loadings ranging from 0.2–3 wt %.

Lamellar thicknesses (*L*) and their relative probabilities can be directly calculated using the DSC endotherms with eq 3 (Gibbs–Thomson equation) and 4. The results for LLDPE_V_-ZnSt and LLDPE_V_-Zn­(acac)_2_ can be seen in Figure 3c,g (Table S3). The smaller lamellar thickness populations correspond to crosslinked fractions, while the larger *L* values represent uncross-linked fractions. The cross-linked lamellae remained consistent, with ZnSt samples measuring 5.9–6.0 nm and Zn­(acac)_2_ samples measuring 5.6–5.9 nm across loadings. Interestingly, the thickest lamellae appeared at 0.5 wt % for both catalysts. In contrast, the uncross-linked fraction showed greater variability, where neat LLDPE had a *L* of 9.1 nm, while LLDPE_v_-ZnSt ranged from 8.3 to 9.3 nm and LLDPE_V_-Zn­(acac)_2_ from 9.6 to 9.9 nm. Gel fraction analysis was conducted to qualitatively assess the yield of the esterification reaction, where an increased gel fraction indicates a greater extent of network formation due to more polymer chains participating in cross-linking. The gel fractions could be determined by removing the uncross-linked fraction using solvent extraction with hot (120 °C) xylenes and eq 1. Notably, this extraction method can also remove some of the catalyst (e.g., ZnSt). After extraction, the remaining material (LLDPE_V_-insol) behaves like a permanent network and cannot be reprocessed. DSC thermograms show a shift in the peak crystallization temperature (*T*_p_) to lower values, indicating less stable crystalline domains (Figure S4a). However, recompounding LLDPE_V_-insol to reintroduce ZnSt (LLDPE_V_-insol-ZnSt 1 wt %) restores processability and shifts *T*_p_s back to higher temperatures (Figure S4b), suggesting that catalyst presence is essential for dynamic bond exchange and thermal stability. The temperatures are given in Table S7. The gel fraction results for the vitrimers with ZnSt and Zn­(acac)_2_ can be found in Figure 3d,h (Table S3). For LLDPE_V_-ZnSt, with increasing catalyst loading, the gel fractions were 62%, 63%, 74%, and 72% and with increasing Zn­(acac)_2_ they were 68%, 73%, 84%, and 82%. These results demonstrate that with increasing catalyst loading the system is able to achieve higher gel fractions. However, the trend is more pronounced with Zn­(acac)_2_, suggesting that it may be able to catalyze the reaction more efficiently. This has been shown in the literature where reports of flow activation energies (*E*_a_), encompassing contributions from bond exchange, chain mobility, and network architecture, for Zn­(acac)_2_ have been lower than ZnSt based counterparts.^75–77^ Returning to the crystallinity (χ_c_) data, both LLDPE_V_-ZnSt and LLDPE_V_-Zn­(acac)_2_ exhibit similar χ_c_ values across most loadings, despite notable differences in gel fraction, up to ∼10%, with the exception of the 0.2 wt % samples. This result suggests that crystallization behavior could be influenced by more complex factors than cross-link density alone. Correlating this data back to the extensibilities of the vitrimers, for LLDPEv-ZnSt, the most extensible sample is at 3 wt %, which also has the lowest crystallinity and thinnest lamellae. A moderate degree of crystallinity combined with thinner lamellae can enhance the extensibility by increasing the mobility of the amorphous phase. Additionally, relatively minor changes in melting temperatures across loadings may suggest a more homogeneous system. For LLDPEv-Zn­(acac)_2_, the most extensible sample is also at 3 wt % of catalyst loading content. This occurs despite a general increase in crystallinity (except the 0.5 wt % point) and gel fraction with increasing catalyst loading, both of which typically contribute to a stiffer material. However, the observed decrease in lamellar thicknesses with the increasing catalyst may promote a more mobile amorphous phase surrounding the crystallites. Furthermore, the increased variability in *T*_m_s could indicate a more heterogeneous system, where the catalyst may preferentially aggregate within the cross-linked fraction. To further understand these complex effects, the crystallization behavior of these distinct vitrimers under nonisothermal crystallization conditions needs to be examined.^53^

The nonisothermal exotherms with cooling rates (ϕ) of 2, 5, 8, 10, 13, 20, and 40 °C/min for neat LLDPE, LLDPE_V_-ZnSt, and LLDPE_V_-Zn­(acac)_2_ can be found in Figure S5. As the cooling rate increases, it is observed that both the onset temperature (*T*_onset_) and peak crystallization temperature (*T*_p_) decrease (temperatures for all samples are listed in Table S7). In addition, it can be observed that the crystallization peaks get broader with increasing cooling rates and is due to the formation of imperfect crystals with more rapid cooling.^54^ Similar to the melting endotherms, all vitrimer sample exotherms are bimodal. The higher temperature crystallization peak is denoted as *T*_p,1_ and the lower temperature as *T*_p,2_. A lower *T*_p_ suggests that a higher degree of supercooling was required due to a decrease in chain mobility. Thus, for the vitrimer samples, *T*_p,1_ is attributed to the uncross-linked fraction and *T*_p,2_ to the crosslinked portion. The nonisothermal crystallization data for LLDPE, LLDPE_V_-ZnSt, and LLDPE_V_-Zn­(acac)_2_ were further analyzed using the Jeziorny-modified Avrami model. This was done by first determining the relative crystallinity as a function of temperature using eq 7 (Figure S6), the crystallization time could then be determined using the temperature and eq 8. Avrami plots could then be made using a rearranged Avrami eq (eq 6) and the plots for LLDPE and the vitrimers with ZnSt and Zn­(acac)_2_ can be found in Figure S7. These plots reveal three distinct regions of crystallization behavior. In the initial stage, crystallization is primarily driven by nucleation with minimal crystal growth, as indicated by slopes much less than 1. The second region represents the main crystallization phase, where both nucleation and crystal growth occur concurrently; this region is the focus of subsequent analysis. The final stage, characterized by slopes much greater than 1, corresponds to the late stages of crystallization, which are not well-described by the Avrami model.

From the Avrami plots, both the Avrami exponent (*n*) and the Avrami constant (*K*) can be determined from the linear region where the Avrami equation is valid, using the slope and y-intercept of the plot (eq 6). The exponent, *n*, reflects the nucleation mechanism, growth dimensionality, and crystal geometry while the constant, *K*, represents the overall crystallization rate. However, since the crystallization was performed under nonisothermal conditions, it is necessary to apply a correction to *K*, yielding the Jeziorny-modified Avrami constant (*K*′), as described by eq 9.^54,58,59^ Previous studies have reported Avrami exponents for LLDPE ranging from 2 to 4, depending on the specific crystallization conditions and sample characteristics.^53,78–80^ The Avrami parameters for the second (main) crystallization phase, namely, the exponent *n*_2_ and the Jeziorny-modified rate constant *K*_2_^′^, for LLDPE_V_-ZnSt and LLDPE_V_-Zn­(acac)_2_ can be found in Figure 4 (Table S7). As shown in Figure 4a,c, *K*_2_^′^ shows a dependence on the cooling rate, where at 2 °C/min all vitrimer samples have a *K*_2_^′^ value of ∼0.2, which increases until 20 °C/min where it starts to plateau around 1.0. Comparing the vitrimer *K*_2_^′^ values to those from neat LLDPE (Figure S8), it can be seen that for both catalysts, the values from the vitrimers are lower than the neat material across all cooling rates with the difference being more pronounced at the slower cooling rates (2–10 °C/min), attributed to cross-linking restricting chain mobility and slowing crystallite growth. However, with faster cooling rates (20–40 °C/min), the *K*_2_^′^ values start to converge, likely due to enhanced supercooling, further limiting chain mobility and favoring nucleation-driven crystallization. The *n*_2_ values for both catalysts remain close to 1 across all cooling rates (Figure 4b,d), regardless of loading content, indicating one-dimensional crystal growth accompanied by heterogeneous nucleation, likely occurring on surfaces or impurities. The *n*_2_ values for neat LLDPE can be found in Figure S8. Vitrimer *n*_2_ values are slightly lowered from neat LLDPE across all cooling rates, likely due to a combination of crosslinking and increased nucleation density that can restrict growth. However, LLDPE *n*_2_ values also remaining at 1 at all cooling rates demonstrate that network formation does not significantly impact the growth geometry. Since crystallization was conducted under nonisothermal conditions, the extracted Avrami parameters *n* and *K* may differ from those obtained under isothermal conditions and do not carry the same physical meaning due to the continuous change in the temperature.^53,81,82^ Still, the overlap in *K*_2_^′^ and *n*_2_ values for both catalyst types and all loadings suggests that the catalysts do not significantly influence the crystal growth mechanism or rate once the networks are formed.

**Figure 4:**
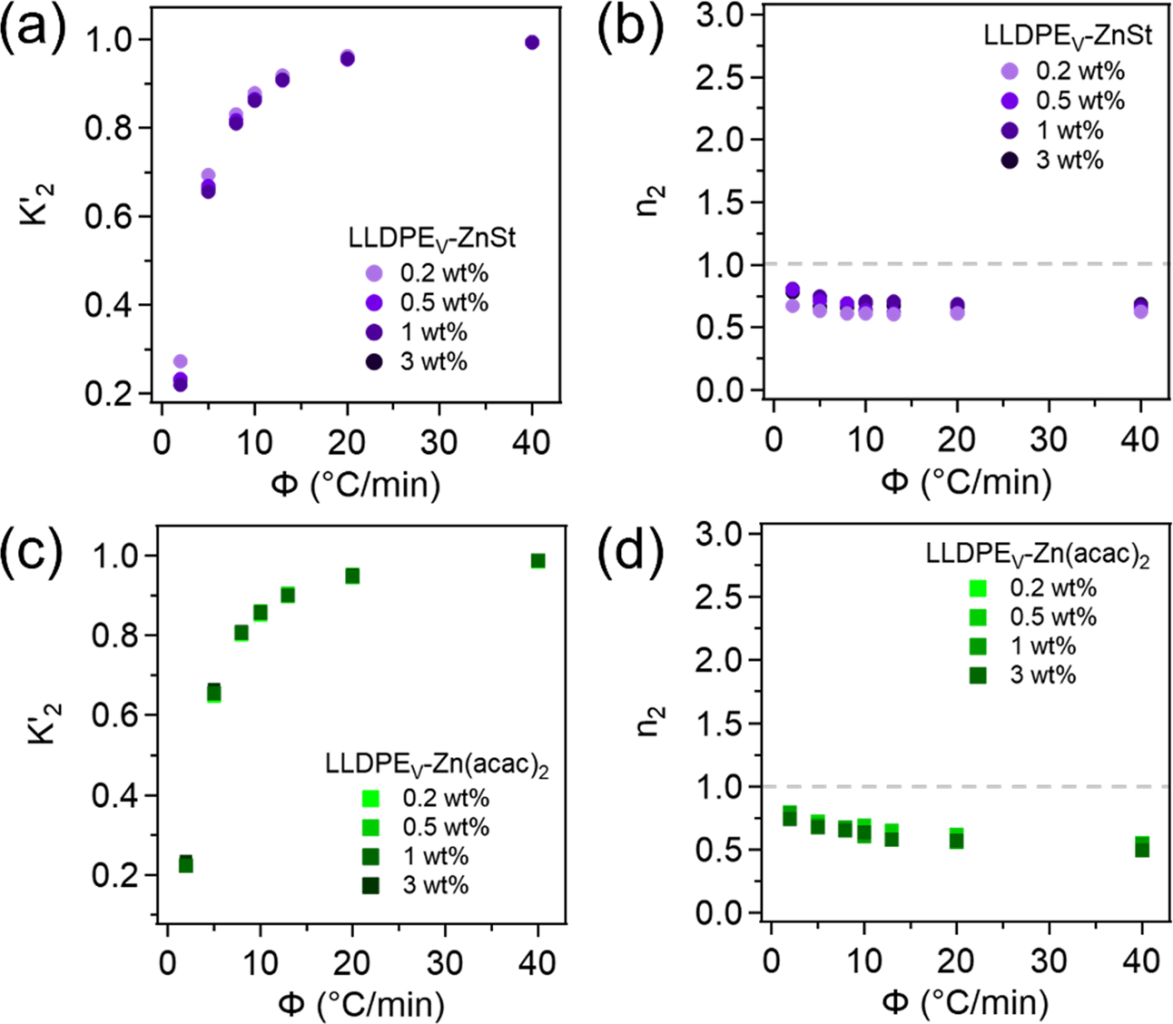
Plots of (a,c) Jeziorny-modified Avrami constant and (b,d) Avrami exponents for LLDPE_V_-ZnSt and LLDPE_V_-Zn­(acac)_2_.

To better understand the nonisothermal crystallization behavior and the influence of catalyst type and loading, the Kissinger method was applied to determine the crystallization activation energy (Δ*E*) for neat LLDPE and vitrimer samples containing ZnSt and Zn­(acac)_2_. This analysis, based on the Kissinger eq (eq 10), utilizes the cooling rates and crystallization peak temperatures (*T*_p_). In general, Δ*E* comprises both the nucleation activation energy (related to the formation of critical crystal nuclei) and the transport activation energy (associated with segmental mobility across the phase boundary to the crystal growth front).^83^ The resulting plots can be found in Figure 5 where the slope can be used to determine the Δ*E*. The Δ*E* values for neat LLDPE and all vitrimers can be found in Table S5. For reference, LLDPE had an Δ*E* value of 337.1 kJ/mol across all cooling rates. Notably, all vitrimer samples showed two distinct regions with different activation energies. For LLDPE_V_-ZnSt, activation energies (Δ*E*) at slow cooling rates (2–13 °C/min) were 557.2, 392.6, 561.4, and 568.3 kJ/mol for 0.2, 0.5, 1, and 3 wt %, respectively, while at fast cooling rates (20–40 °C/min) they were 247.5, 155.9, 180.1, and 211.1 kJ/mol. For LLDPE_V_-Zn­(acac)_2_, slow-rate Δ*E*s were 530.1, 599.6, 794.0, and 767.7 kJ/mol and fast-rate values were 162.5, 171.0, 270.5, and 274.5 kJ/mol, respectively, for the same loadings. This behavior suggests that both ZnSt and Zn­(acac)_2_ likely act as nucleating agents. At faster cooling rates, crystallization appears nucleation-dominated, with the catalysts potentially lowering the overall Δ*E* by reducing the nucleation energy barrier. In contrast, at slower cooling rates, crystal growth becomes the dominant mechanism, and the dynamic cross-links increasingly hinder chain mobility, raising the transport activation energy and thus Δ*E*.

**Figure 5:**
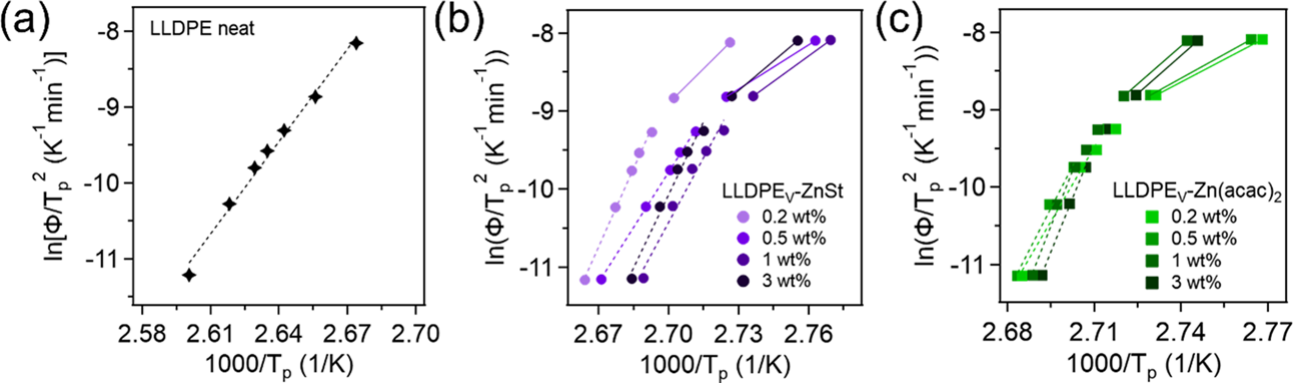
Kissinger plots of (a) LLDPE, (b) LLDPE_v_-ZnSt, and (c) LLDPE_v_-Zn­(acac)_2_. For the vitrimers, these results are derived using *T*_p,2_, the crystallization peak for the cross-linked fraction.

Control samples of physical blends of LLDPE with ZnSt or Zn­(acac)_2_ were formulated and investigated with the same loadings as the vitrimers, 0.2, 0.5, 1, and 3 wt % (with respect to the mass of LLDPE). The FTIR spectra for the blends with a catalyst loading of 1 wt % can be found in Figure S9. Here, the characteristic PE bands can still be seen, including the MA bands at 1790 and 1712 cm^–1^ while no ester bands are present. The melting endotherms for all of the blends can be seen in Figure S10, and the melting temperatures and resulting lamellar thicknesses are listed in Table S3. The addition of ZnSt formed slightly thinner lamellae than neat LLDPE, ranging from 8.6–9.0 nm. Alternatively, Zn­(acac)_2_ had a larger impact on lamellar size and had *L*s of 9.0, 9.0, 10 nm for 0.2, 0.5, and 1 wt %, respectively, while the 3 wt % had two size populations of 6.0 and 8.8 nm (discussed further below). The χ_c_ values with respect to catalyst loading for both ZnSt and Zn­(acac)_2_ can be seen in Figure 6a,c, respectively. For blends with ZnSt, having a higher loading does not heavily impact χ_c_ and stays consistent at ∼40%, all above the neat LLDPE (39.3%). Conversely, adding more Zn­(acac)_2_ causes χ_c_ to decrease (after an initial increase) and has values of 39.6%, 40.0%, 38.4%, and 33.0% with increasing loadings.

**Figure 6:**
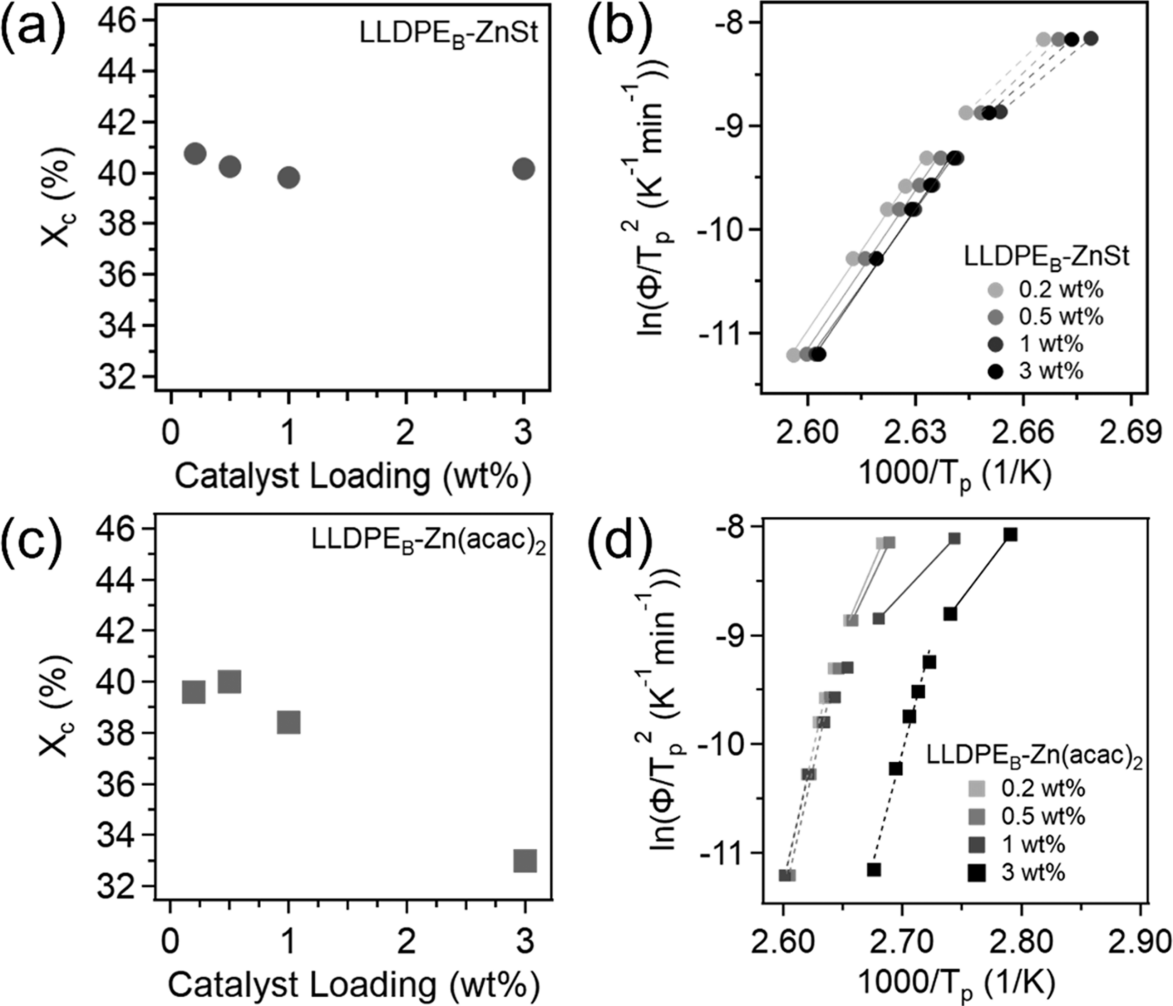
(a,c) Plot of χ_c_ and (b,d) Kissinger plot for LLDPE_B_-ZnSt and LLDPE_B_-Zn­(acac)_2_.

The nonisothermal crystallization behaviors of these blends were further studied as shown in Figure S11 (DSC exotherms). Notably, all blends exhibit a single, sharp crystallization peak, except those containing Zn­(acac)_2_ at 1 and 3 wt % loadings. At 1 wt %, the peak becomes significantly broadened, and at 3 wt %, this broadening remains and develops into a bimodal profile. This behavior is indicative of a mixed-component system, likely resulting from localized regions of a higher Zn­(acac)_2_ concentration, possibly suggesting catalyst aggregation. For this sample (LLDPE_B_-Zn­(acac)_2_ 3 wt %), the higher crystallization temperature, *T*_p,1_, is attributed to the fraction with a lower concentration of Zn­(acac)_2_ (*L* = 8.8 nm) and *T*_p,2_ to the fraction with a higher concentration of Zn­(acac)_2_ (*L* = 6.0 nm). The Jeziorny–Avrami model was also applied to analyze the kinetics and mechanisms of crystal growth in the blends. The *X*(*t*) and Avrami plots for all of the blends can be found in Figures S12 and S13, respectively. The Avrami exponents and constants were determined using eq 6 and their plots can be found in Figure S14. In Figure S14a, with ZnSt, it is observed that the loading level has nearly no impact on the *K*_2_^′^ and *n*_2_ values where *K*_2_^′^ still plateaus at 1.0 at 20 °C/min and *n*_2_ remains at approximately 1. Similar results are seen for Zn­(acac)_2_ in Figure S14b, except for *K*_2_^′^, both the 1 and 3 wt % show slightly decreased values in the range of 2–20 °C/min cooling range and the 3 wt % also shows slightly lowered *n*_2_ values across all cooling rates. Kissinger plots for ZnSt and Zn­(acac)_2_ blends are shown in Figure 6b,d, revealing two distinct kinetic regimes with different activation energies (Table S6). Across all blend samples, activation energies in the slower cooling rate region (2 to 13 °C/min) show minimal variation within each catalyst type. In contrast, activation energies in the faster cooling rate region (20–40 °C/min) show a decreasing trend with increased catalyst loading levels. Blends with ZnSt have activation energies of 274.3, 274.4, 235.3, and 258.8 kJ/mol with increasing loadings. The decreasing trend is more pronounced with Zn­(acac)_2_ blends with increased loading levels and the values were 217.4, 191.1, 97.3, and 120.6 kJ/mol.

Figure 7 illustrates these trends of the catalysts’ increasing Δ*E* during the slower cooling rates (2–13 °C/min) while decreasing Δ*E* when fast cooling rates are used (20–40 °C/min) to highlight the different behaviors in the vitrimers and physical blends. In Figure 7a (slow cooling, ZnSt), catalyst loading has little effect on the blend activation energies. The ∼150 kJ/mol increase in the vitrimer crystallization activation energies is attributed to crosslinking, which restricts chain mobility and raises the energy required for segmental transport to the crystal front. Figure 7b (fast cooling, ZnSt) shows similarly stable activation energies in blends, while vitrimer values increase with loading after an ∼100 kJ/mol drop at 0.5 wt %, likely due to increased crosslinking evidenced in the gel fraction data. Across all catalyst loadings within this cooling rate region, the vitrimers exhibit reduced activation energies compared to the uncrosslinked blends. This reduction is attributed to the increased presence of catalysts acting as nucleating agents, which shortens the distance chains must travel to reach a crystal face. This effect is especially pronounced in the presence of crosslinks, which further constrains chain mobility and enhances the nucleation efficiency. Figure 7c (slow cooling, Zn­(acac)_2_) shows that blend activation energies remain relatively constant, while the value of vitrimers increases with catalyst loading, again attributed to crosslinking effects. At low catalyst loadings (0.2 and 0.5 wt %), the difference in activation energies between the vitrimers and blends is relatively small for Zn­(acac)_2_ compared to ZnSt. This may reflect the superior nucleating ability of Zn­(acac)_2_ at low concentrations, before the inhibitory effects of crosslinking begin to dominate at higher loadings. Interestingly, Figure 7d (fast cooling, Zn­(acac)_2_) reveals opposite trends, where vitrimer activation energies increase with loading, while blend values decrease. This suggests that Zn­(acac)_2_ promotes nucleation in blends, continually lowering the energy barrier, while cross-link formation in the vitrimers raises it.

**Figure 7:**
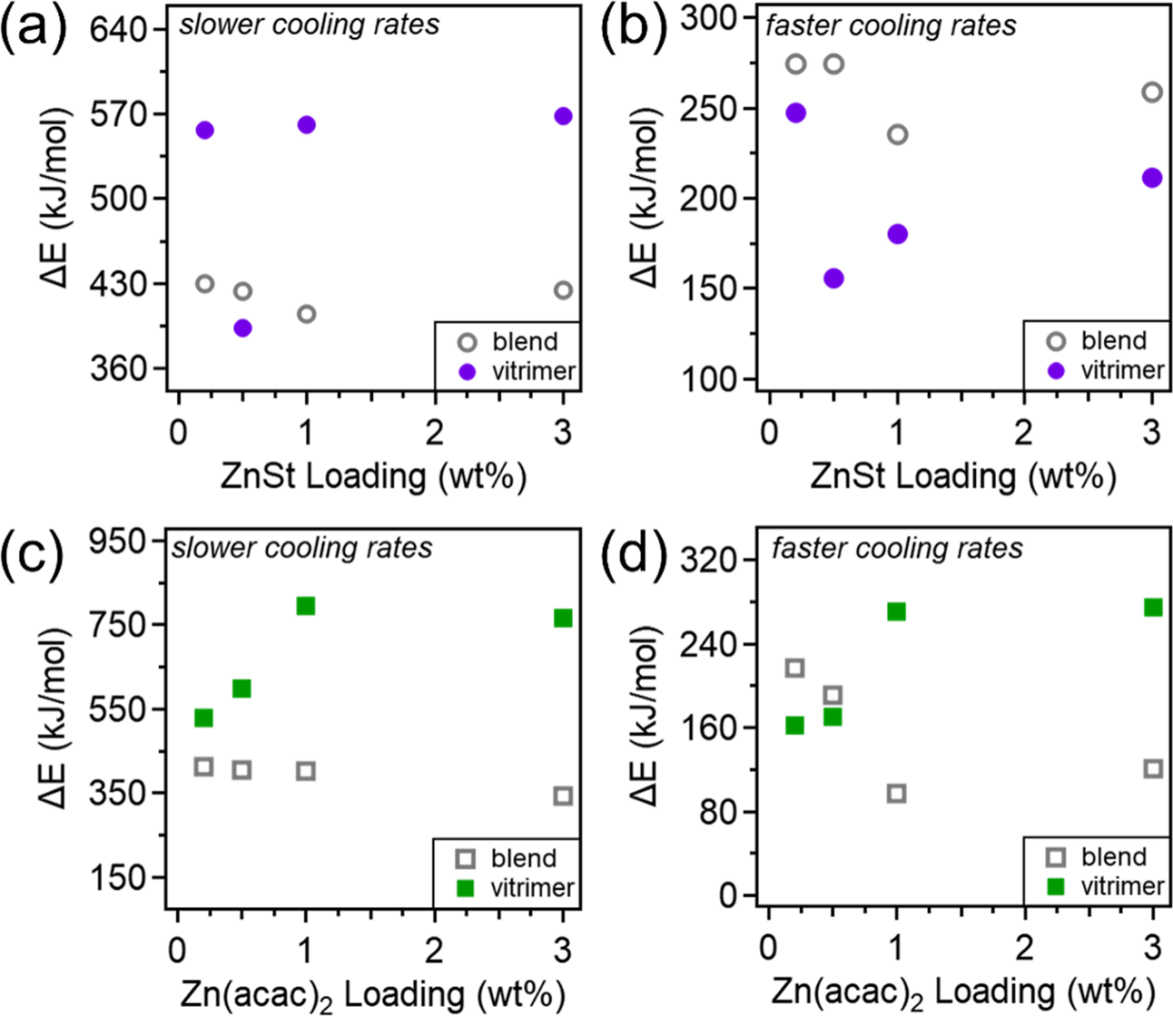
Plot of crystallization activation energies for vitrimers and blends with (a,b) ZnSt and (c,d) Zn­(acac)_2_ where slower cooling rates range from 2–13 °C/min and the faster cooling rates range from 20–40 °C/min.

Altogether, theis data highlights key differences in how ZnSt and Zn­(acac)_2_ influence dynamic networks formed from LLDPE and DGEBA, particularly in the context of the interplay between dynamic crosslinking and crystallization. Zn­(acac)_2_ promotes higher gel fractions but correspondingly reduces the achievable crystalline fraction. In contrast, ZnSt has a higher flow activation energy, resulting in reduced gel fractions, while allowing for higher χ_c_ values. Both catalysts show evidence of nucleating behavior in vitrimers and physical blends. Zn­(acac)_2_ appears to lower the crystallization energy barrier more effectively than ZnSt, but this can lead to crystallite crowding, which limits the final crystallinity. Additionally, Zn­(acac)_2_ appears to catalyze esterification reactions more efficiently, particularly in localized regions of the network. This is supported by Figure S15, which presents Kissinger plots for *T*_p,1_ (uncross-linked fraction) of ZnSt and Zn­(acac)_2_ vitrimers. ZnSt shows two distinct activation energy regions, indicating nucleating agent presence in both cross-linked and uncrosslinked regions. In contrast, Zn­(acac)_2_ displays a single activation energy across all cooling rates in the uncrosslinked fraction (i.e., no nucleating agent present), suggesting it significantly enhances crosslinking. This behavior likely stems from the long alkyl chain on ZnSt, which integrates well into the PE matrix. These effects may be further influenced by secondary structures: ZnSt can form multinuclear aggregates with Zn–O cores and micelle-like alkyl shells, increasing its “solubility” in PE while potentially limiting its catalytic and nucleating efficiency.^84–86^ Zn­(acac)_2_, typically trimeric in the solid state, likely becomes monomeric in the melt. This monomeric state may enhance its catalytic and nucleating roles by having the carbonyls that catalyze esterification reactions more accessible while the more polar structure could disrupt the PE melt more efficiently, causing crystal nucleation.^87^ Overall, the increased crystallinity and more moderate gel fractions of ZnSt-based vitrimers may contribute to their enhanced mechanical properties.

To further extend the study, vitrimers and physical blends were also made using zinc­(II) acetate (Zn­(ac)_2_) and manganese­(II) acetate (Mn­(ac)_2_), all with catalyst loadings of 1 wt %, with respect to the mass of LLDPE. Sample FTIR spectra (Figure S9), crystallization exotherms with the same cooling rates as previously mentioned (Figure S17), *X*(*t*) plots (Figure S18), Avrami plots (Figure S19), and plots of *K*_2_^′^ and *n*_2_ (Figure S20) along with blend melt endotherms (Figure S10) and vitrimer DMA and tensile properties (Figure S16 and Table S4) can be found in the Supporting Information. The FTIR and DMA data show that both Zn­(ac)_2_ and Mn­(ac)_2_ form networks and have rubbery moduli of 1.46 and 1.12 MPa, respectively. The gel fractions were determined to be 74% (Zn­(ac)_2_) and 71% (Mn­(ac)_2_). The melt endotherms for both vitrimers are listed in Figure 8a. Both show bimodal behavior, and PE_V_-Zn­(ac)_2_ has *T*_m_s of 111.6 and 122.3 °C and PE_V_-Mn­(ac)_2_ has *T*_m_s of 111.9 and 122.5 °C. These temperatures correspond to *L*s of 5.8 and 9.0 nm for Zn­(ac)_2_ and 5.9 and 9.1 nm for Mn­(ac)_2_ and the χ_c_s were found to be 32.2% (Zn­(ac)_2_) and 33.4% (Mn­(ac)_2_). Collectively, showing that Zn­(ac)_2_ is likely to have a lower exchange activation energy (previously reported in a range of ∼50–86 kJ/mol)^19,76,77^ than Mn­(ac)_2_, leading to the greater gel fraction. These properties are reflected in the tensile data, where the vitrimer made with Mn­(ac)_2_ has a higher modulus (116.4 MPa), intermediate to those for ZnSt and Zn­(acac)_2_ at 1 wt %, and for strain at break and toughness, samples prepared using Mn­(ac)_2_, Zn­(ac)_2_, and Zn­(acac)_2_ are all similar.

**Figure 8:**
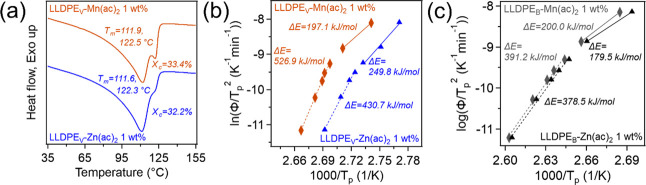
(a) DSC thermograms showing melting endotherms and χ_c_ and (b) Kissinger plot for PE_V_-Zn­(ac)_2_ and PE_V_-Mn­(ac)_2_. (c) Kissinger plot of PE_B_-Zn­(ac)_2_ and PE_B_-Mn­(ac)_2_.

Results from the Jeziorny-modified Avrami analysis again show that catalyst type has little effect on the *K*_2_^′^ and *n*_2_ values for both the vitrimers and blends (Figure S20). The Kissinger plots for the vitrimers created with Zn­(ac)_2_ and Mn­(ac)_2_ are shown in Figure 8b. Here, two distinct regions dominated by nucleation and growth are observed. Mn­(ac)_2_ appears to be the more effective nucleating agent, as indicated by its lower activation energy in the fast cooling regime (197.1 vs 249.8 kJ/mol). In the slow cooling regime, however, Mn­(ac)_2_ shows a higher activation energy (526.9 vs 430.7 kJ/mol), which is likely due to crystallite crowding where excessive nucleation leads to spatial constraints that hinder crystal growth. Despite this, LLDPE_V_–Mn­(ac)_2_ achieves a higher χ_c_, attributed to its lower gel fraction and reduced cross-link density, allowing for more extensive crystal growth. Kissinger plots for the physical blends can be found in Figure 8c. Moreover, two distinct crystallization regimes are observed, further confirming that both Zn­(ac)_2_ and Mn­(ac)_2_ can function as nucleating agents. Δ*E* for both catalysts are very similar in each regime, 200.0 vs. 179.5 kJ/mol in the nucleation-dominated region and 391.2 vs. 378.5 kJ/mol in the growth-dominated region, indicating that Zn­(ac)_2_ and Mn­(ac)_2_ behave similarly to each other, and also to Zn­(acac)_2_, which was anticipated given their closely related chemical structures. The decreased gel fractions and increased crystallinity observed in vitrimers containing Zn­(ac)_2_ and Mn­(ac)_2_, relative to those with Zn­(acac)_2_, are likely due to the ability of Zn­(ac)_2_ and Mn­(ac)_2_ to form coordinated aggregates.^88^ These aggregates may reduce the catalytic and nucleating efficiency of the metal salts by limiting their accessibility and mobility within the polymer network.

Although industrial cooling rates, often reaching hundreds to thousands of °C/min, far exceed those achievable with conventional DSC techniques, the results from these LLDPE-derived vitrimers in this study suggest that at faster cooling rates (>20 °C/min), the effects of network formation and catalyst presence on crystallization rates become less pronounced. This is evidenced by the convergence of parameters such as *K*_2_^′^ and *t*_1/2_ (Table S7) across all samples. However, variations in transesterification catalyst type and loading still significantly influence the final crystallinity, extent of crosslinking, and mechanical properties. Given that the semicrystalline nature of PE (and polyolefins in general) is central to its desirable properties, it remains important to further investigate how these factors and processing conditions interact. Overall, the findings in this work indicate that dynamic bonding can be introduced to enable reprocessability without substantially compromising crystallization behavior under practical processing conditions and that catalyst selection and loading content offer a promising route for fine-tuning material performance.

## Conclusion

This study systematically investigates the role of transesterification catalyst identity and loading content on the nonisothermal crystallization behavior of LLDPE-derived vitrimers as well as their mechanical properties. We demonstrate that catalyst selection can significantly influence the gel content, degree of crystallinity, crystal lamellar size, and crystallization kinetics. Zn­(acac)_2_, Zn­(ac)_2_, and Mn­(ac)_2_ promote higher gel fractions and exhibit strong nucleating behavior but also introduce reduced crystallinity. In contrast, ZnSt yields higher crystallinity and improved mechanical performance, likely due to the more moderate gel content with thinner lamellae. Jeziorny-modified Avrami and Kissinger analyses reveal distinct nucleation- and growth-dominated regimes, with activation energies sensitive to both catalyst type and the cooling rate. Importantly, at faster cooling rates (>20 °C/min), crystallization kinetics converge across all systems, suggesting that incorporation of dynamic bonds would not significantly compromise processability. These findings provide valuable design principles for tailoring semicrystalline vitrimer networks and highlight catalyst selection as a key parameter for optimizing performance in sustainable polymer systems.

## Supplementary Material



## Data Availability

The data supporting this study are available upon request from the corresponding author.

## Notes

FTIR spectra, DMA storage modulus–temperature plots, table listing rubbery modulus, stress–strain curves from uniaxial tension, table listing tensile properties (modulus, ultimate tensile strength, strain at break, and toughness), DSC crystallization exotherms, development of crystallinity plots, Avrami plots, Avrami exponent and Jeziorny-modified constant plotted against cooling rates, DSC melting endotherms, table listing *T*_m_, χ_c_, *L*, and gel fraction, Kissinger plots using *T*_p,1_, table listing Δ*E*, and table listing *T*_onset_, *T*_p_, *t*_1/2_, *K*_2_^′^, and *n*_2_ with varying cooling rates for all samples (PDF)
