# Supplementary material for: Catalyst Influence on the Crystallization Behaviors and Mechanical Properties of Polyethylene Vitrimers
Source: Macromolecules. 2025 Oct 27;58(21):11803–15. doi: 10.1021/acs.macromol.5c02354 (PMC12613809; doi:10.1021/acs.macromol.5c02354)
Supplement: Supplementary file 1 [file ma5c02354_si_001.pdf]

Supporting Information for:

Catalyst influence on the crystallization behaviors and mechanical properties of polyethylene vitrimers

*Sara Valdez and Zhe Qiang**

School of Polymer Science and Engineering, The University of Southern Mississippi, Hattiesburg, MS 39406

Corresponding author: Zhe Qiang Email: zhe.qiang@usm.edu

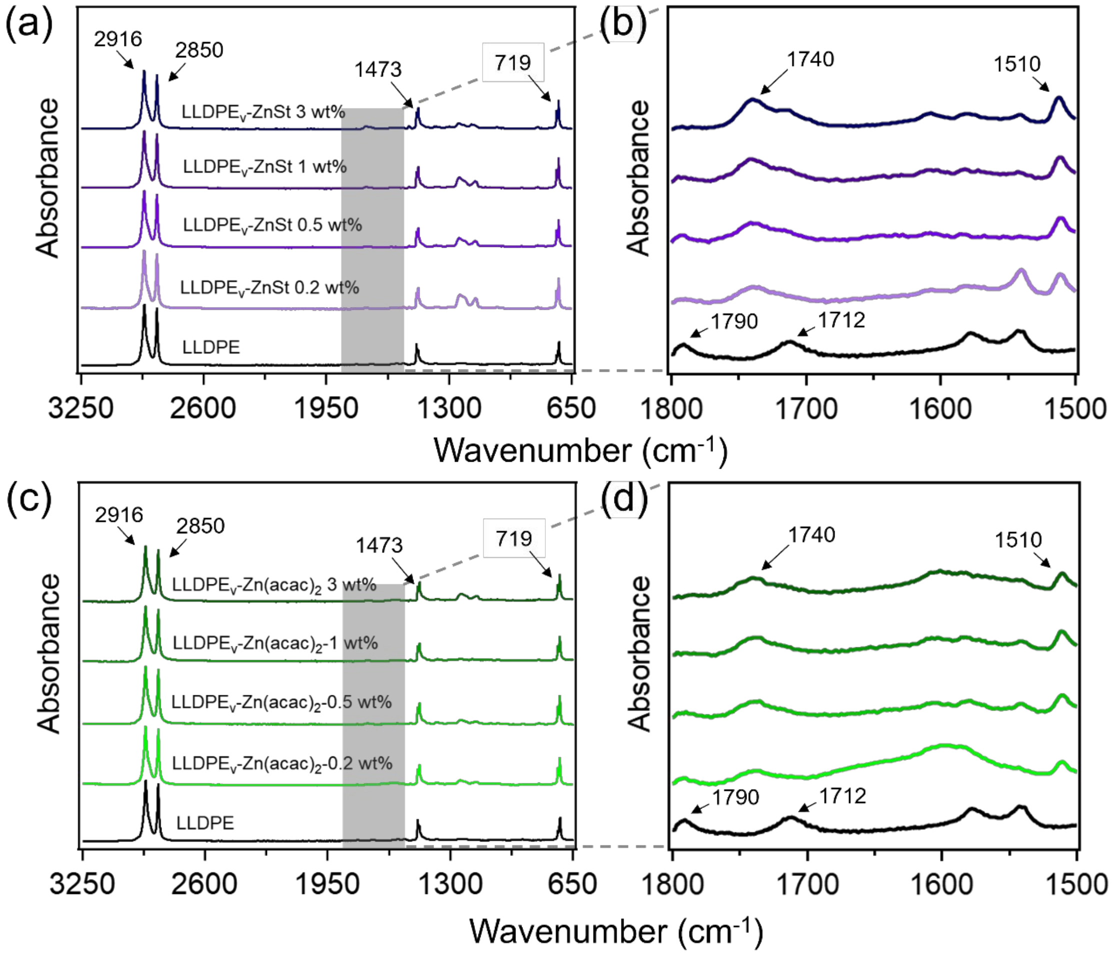

Figure S1. (a, c) FTIR spectra (normalized to the C-H band at 2916 cm^{-1}) and (b, d) closeup of 1500-1800 cm^{-1}, highlighting the area showing the esterification reaction of LLDPEv-ZnSt and LLDPEv-Zn(acac)$_2$ with catalyst loadings ranging 0.2-3 wt%.

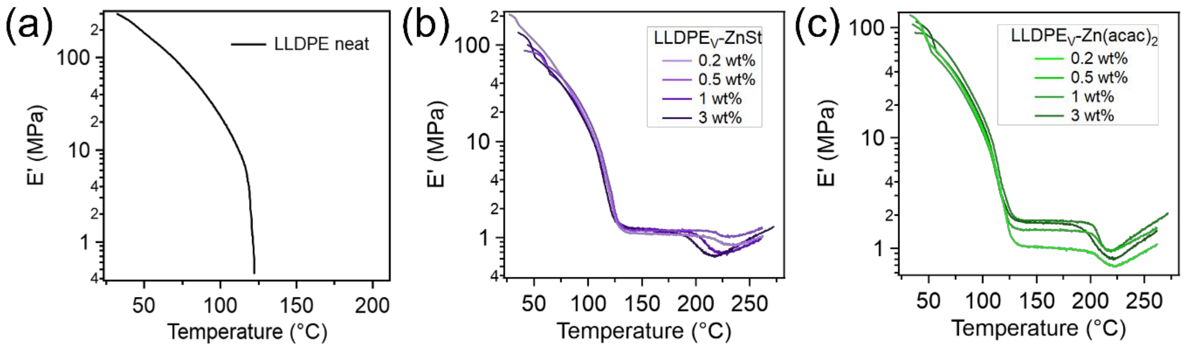

Figure S2. Storage modulus-temperature plots for (a) neat LLDPE (b) LLDPE$_V$-ZnSt, and (c) LLDPE$_V$-Zn(acac)$_2$ with various catalyst loadings.

Table S1. Table listing storage modulus at the rubbery plateau for LLDPE$_V$-ZnSt and LLDPE$_V$-Zn(acac)$_2$.

Sample	Catalyst loading (wt%)	*E'* (MPa)
LLDPE$_V$-ZnSt	0.2	1.12
	0.5	1.18
	1	1.27
	3	1.25
LLDPE$_V$-Zn(acac)$_2$	0.2	1.06
	0.5	1.50
	1	1.81
	3	1.74

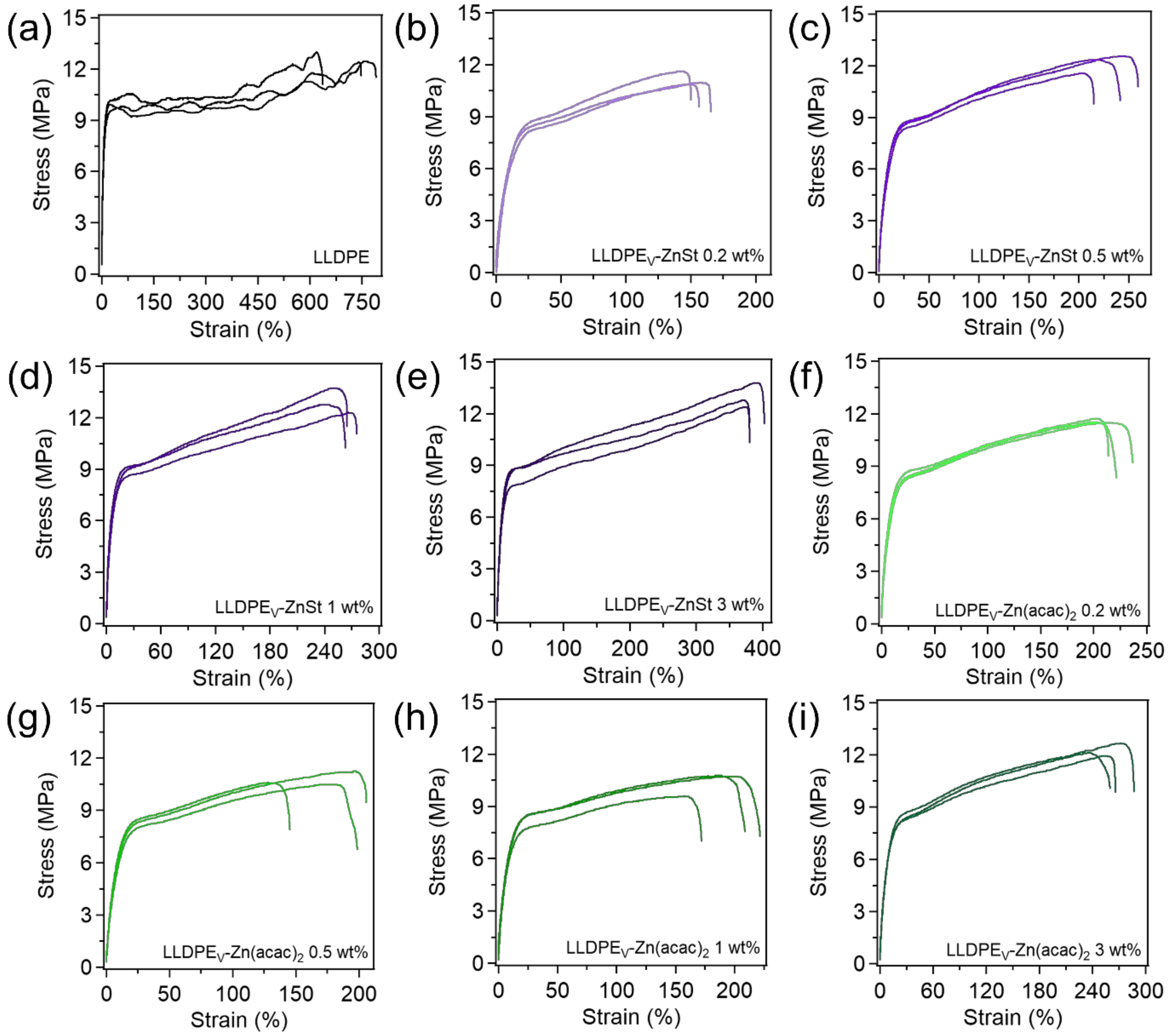

Figure S3. Stress-strain curves under uniaxial tension for (a) LLDPE, (b-e) LLDPE$_V$-ZnSt, and (f-i) LLDPE$_V$-Zn(acac)$_2$ with various catalyst loadings.

Table S2. Table listing tensile properties for LLDPE, $LLDPE_V$-ZnSt, and $LLDPE_V$-$Zn(acac)_2$ including the average (AVG) and standard deviation (STD).

Sample	Run	Modulus (MPa)	UTS (MPa)	Strain at break (%)	Toughness (MJ/m^3)
LLDPE	1	172.6	13.0	636.9	68.83
	2	123.7	12.4	747.3	75.32
	3	146.6	12.4	790.1	82.48
	AVG	*147.6*	*12.6*	*724.8*	*75.48*
	STD	*19.98*	*0.283*	*64.54*	*5.655*
$LLDPE_V$-ZnSt 0.2 wt%	1	90.98	11.6	150.2	14.48
	2	84.41	10.9	156.5	14.24
	3	94.66	10.9	165.3	15.44
	AVG	*90.02*	*11.1*	*157.3*	*14.72*
	STD	*4.239*	*0.349*	*6.193*	*0.5186*
$LLDPE_V$-ZnSt 0.5 wt%	1	81.12	11.6	215.2	20.99
	2	89.19	12.6	259.6	27.35
	3	106.2	12.4	241.7	25.16
	AVG	*92.17*	*12.2*	*238.8*	*24.50*
	STD	*10.45*	*0.434*	*18.24*	*2.638*
$LLDPE_V$-ZnSt 1 wt%	1	134.3	12.3	275.5	28.17
	2	130.3	13.7	264.7	29.45
	3	157.0	12.7	262.9	28.40
	AVG	*140.5*	*12.9*	*267.7*	*28.67*
	STD	*11.76*	*0.592*	*5.564*	*0.5572*
$LLDPE_V$-ZnSt 3 wt%	*1*	127.3	12.8	380.4	39.85
	2	108.5	12.4	380.4	37.41
	3	110.1	12.8	402.5	44.71
	AVG	*115.3*	*12.7*	*387.8*	*40.66*
	STD	*8.510*	*0.170*	*10.42*	*3.034*
$LLDPE_V$-$Zn(acac)_2$ 0.2 wt%	1	84.14	11.7	214.0	21.16
	2	118.6	11.5	221.5	22.05
	3	96.77	11.5	236.7	23.42
	AVG	*99.84*	*11.6*	*224.1*	*22.21*
	STD	*14.23*	*0.106*	*9.443*	*0.9296*
$LLDPE_V$-$Zn(acac)_2$ 0.5 wt%	1	96.81	11.2	206.0	19.81
	2	90.95	10.5	199.0	17.94
	3	104.6	10.6	145.4	13.27
	AVG	*97.45*	*10.8*	*183.5*	*17.00*
	STD	*5.591*	*0.335*	*27.07*	*2.750*

Sample	Run	Modulus (MPa)	UTS (MPa)	Strain at break (%)	Toughness (MJ/m^3)
$LLDPE_V$-$Zn(acac)_2$ 1 wt%	1	94.05	9.60	172.1	14.55
	2	95.66	10.7	221.7	20.92
	3	88.11	10.8	208.9	19.77
	AVG	*92.61*	*10.4*	*200.9*	*18.41*
	STD	*3.247*	*0.542*	*21.02*	*2.772*
$LLDPE_V$-$Zn(acac)_2$ 3 wt%	1	98.75	12.7	287.2	30.50
	2	94.38	12.1	260.0	26.55
	3	96.51	12.0	266.1	26.62
	AVG	*96.55*	*12.3*	*271.1*	*27.89*
	STD	*1.784*	*0.312*	*11.65*	*1.846*

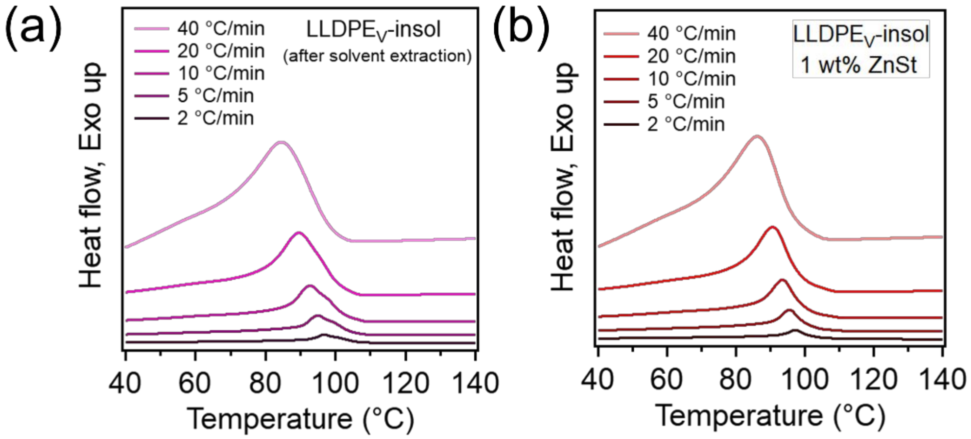

Figure S4. DSC thermograms with varied cooling rates for (a) $LLDPE_V$-insol and (b) $LLDPE_V$-insol-ZnSt 1 wt%.

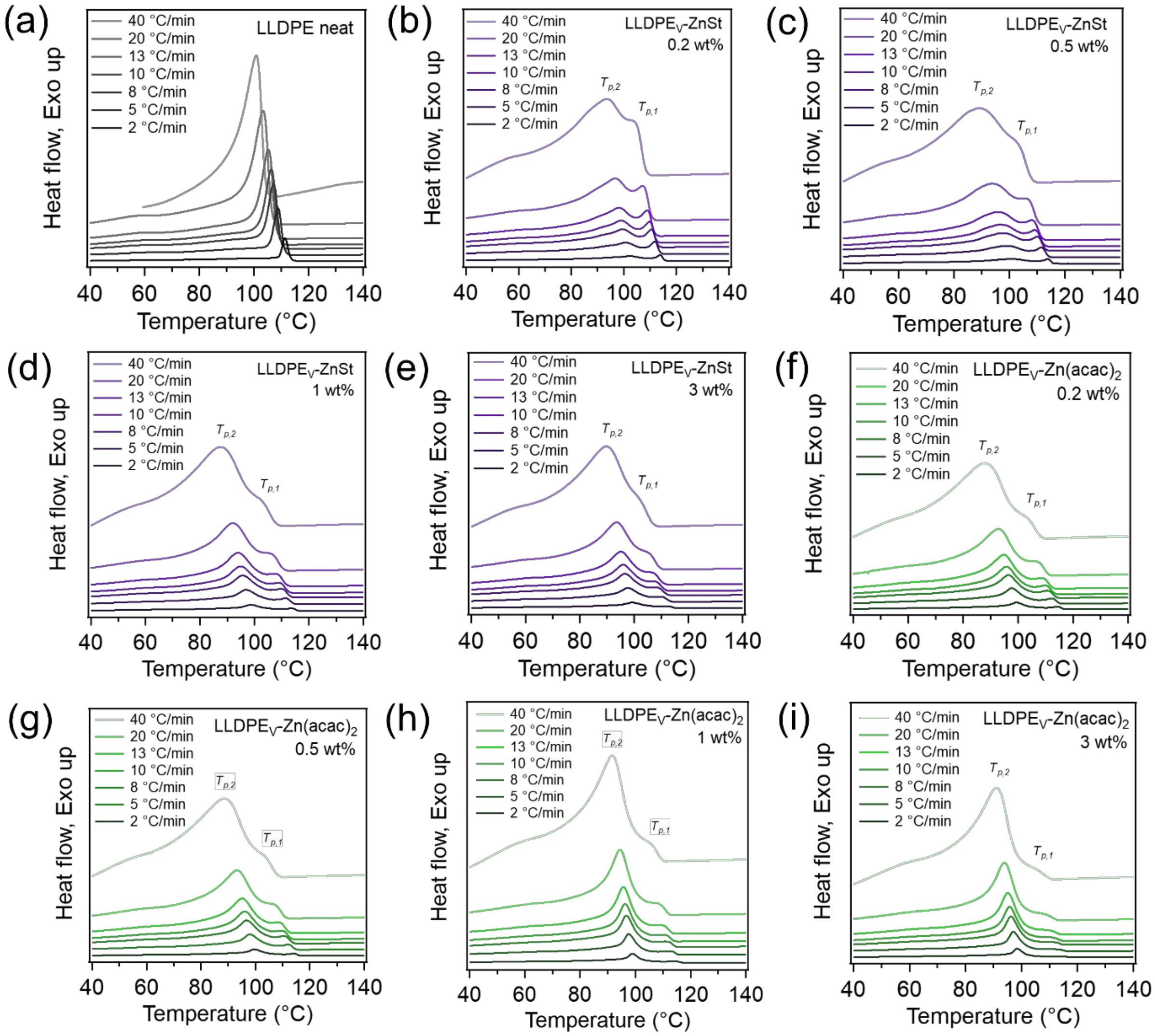

Figure S5. DSC thermograms with varied cooling rates for (a) LLDPE, (b-e) LLDPE$_V$-ZnSt, and (f-i) LLDPE$_V$-Zn(acac)$_2$ with various catalyst loadings.

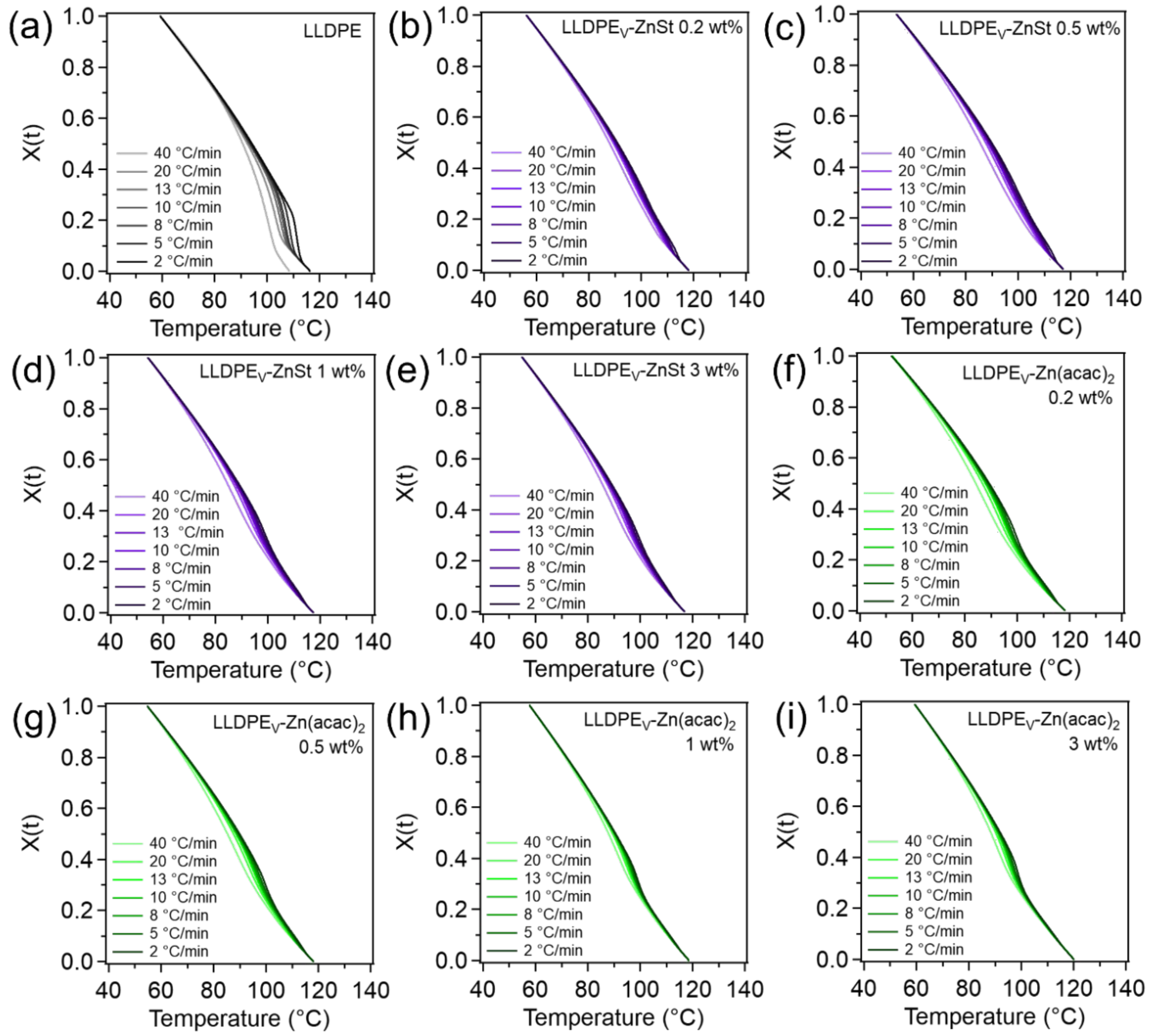

Figure S6. Development of crystallinity with varied cooling rates for (a) LLDPE, (b-e) $LLDPE_V$-ZnSt, and (f-i) $LLDPE_V$-$Zn(acac)_2$ with various catalyst loadings.

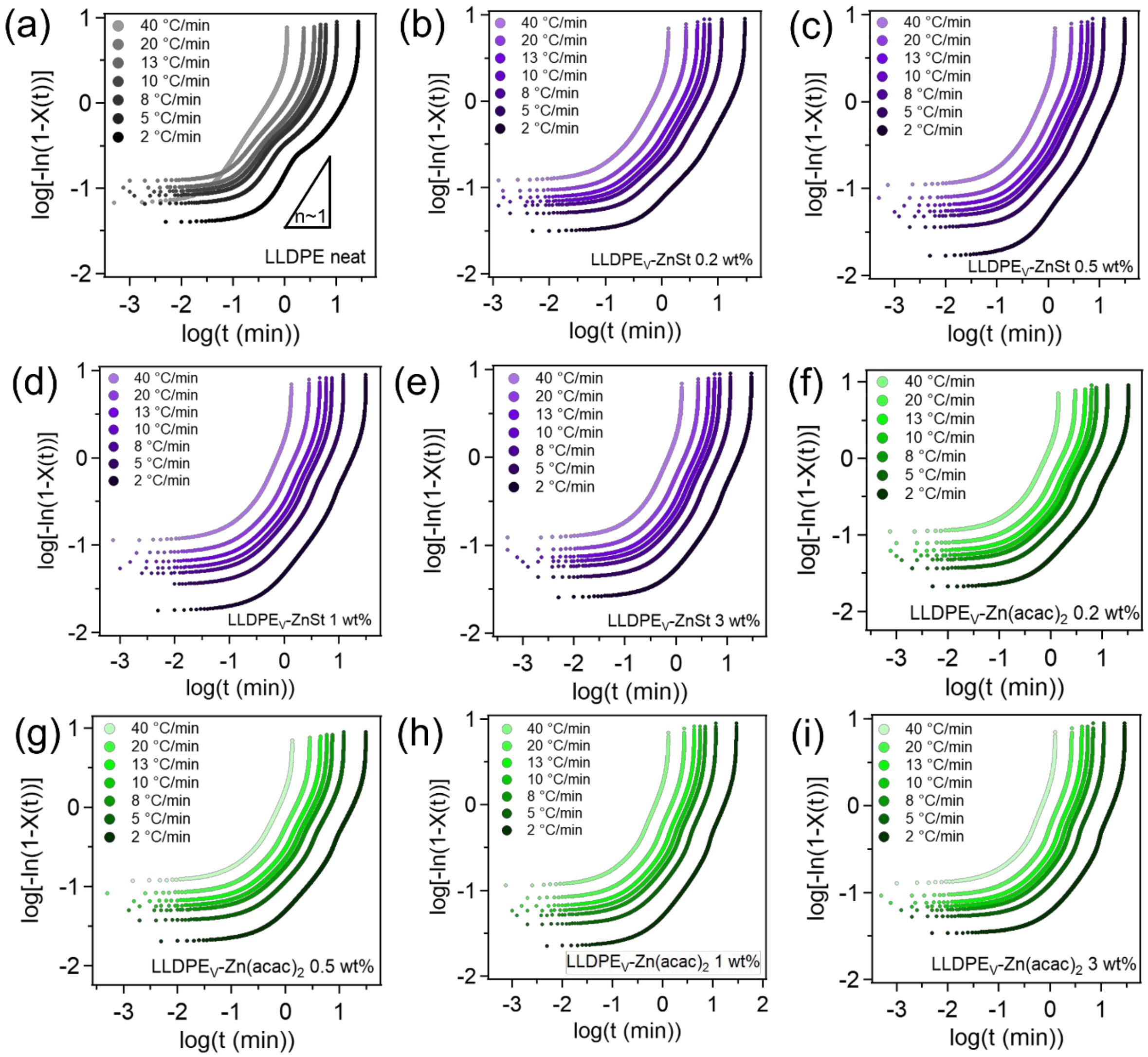

Figure S7. Avrami plots with various cooling rates for (a) LLDPE, (b-e) LLDPE$_V$-ZnSt, and (f-i) LLDPE$_V$-Zn(acac)$_2$ with various catalyst loadings.

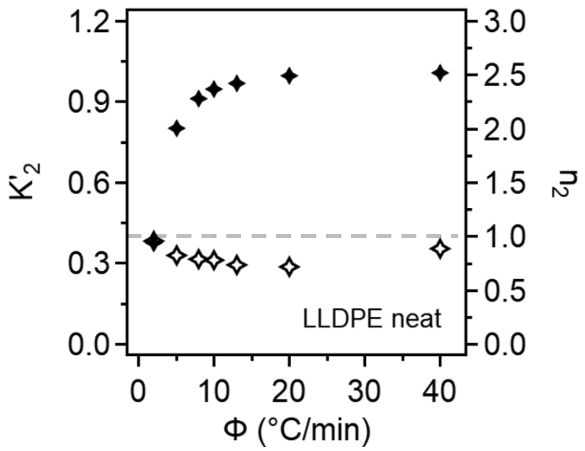

Figure S8. Avrami parameters for neat LLDPE.

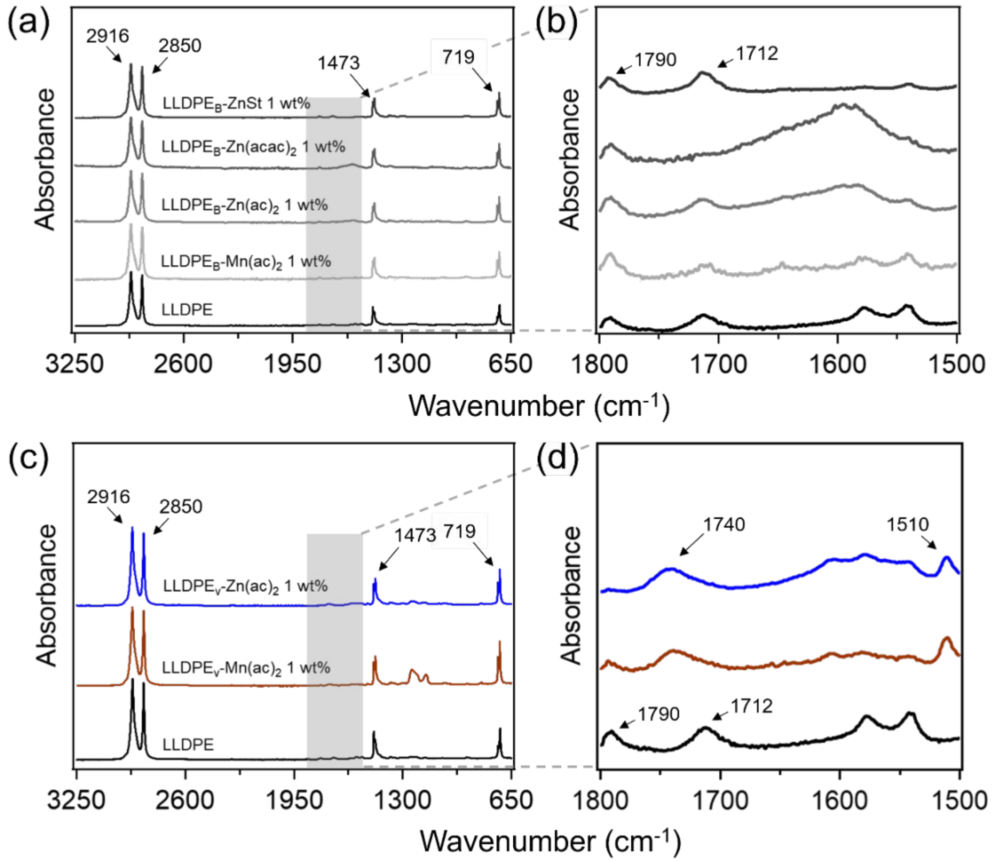

Figure S9. (a, c) FTIR spectra (normalized to the C-H band at 2916 cm^{-1}) and (b, d) closeup of 1500-1800 cm^{-1}, highlighting the area showing the (a, b) lack of reaction for blends with ZnSt, $Zn(acac)_2$, $Zn(ac)_2$, and $Mn(ac)_2$ and (c, d) presence of esterification reaction for vitrimers with $Zn(ac)_2$ and $Mn(ac)_2$ all with a loading of 1 wt%.

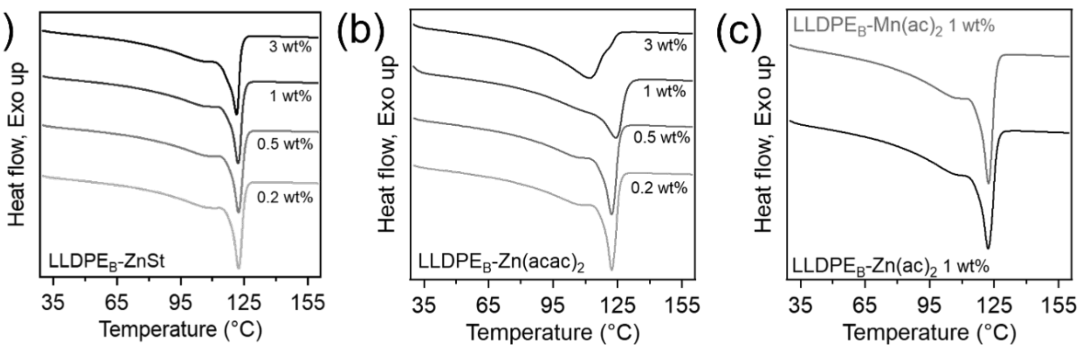

Figure S10. DSC thermograms showing melting for (a) LLDPE$_B$-ZnSt, and (b) LLDPE$_B$-Zn(acac)$_2$, and (c) LLDPE$_B$-Zn(ac)$_2$ and LLDPE$_B$-Mn(ac)$_2$ with various catalyst loadings.

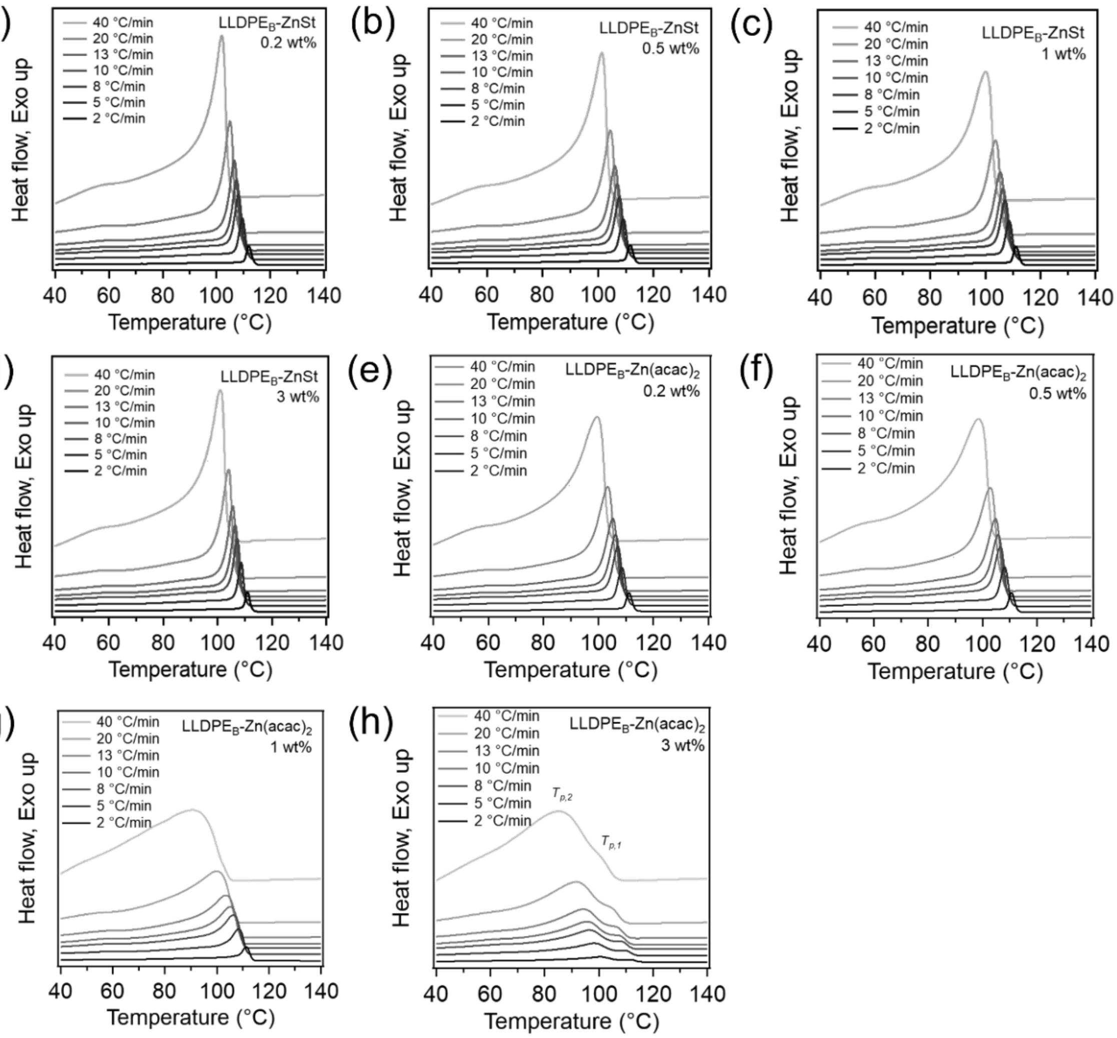

Figure S11. DSC thermograms with varied cooling rates for (a-d) LLDPE$_B$-ZnSt, and (e-h) LLDPE$_B$-Zn(acac)$_2$ with various catalyst loadings.

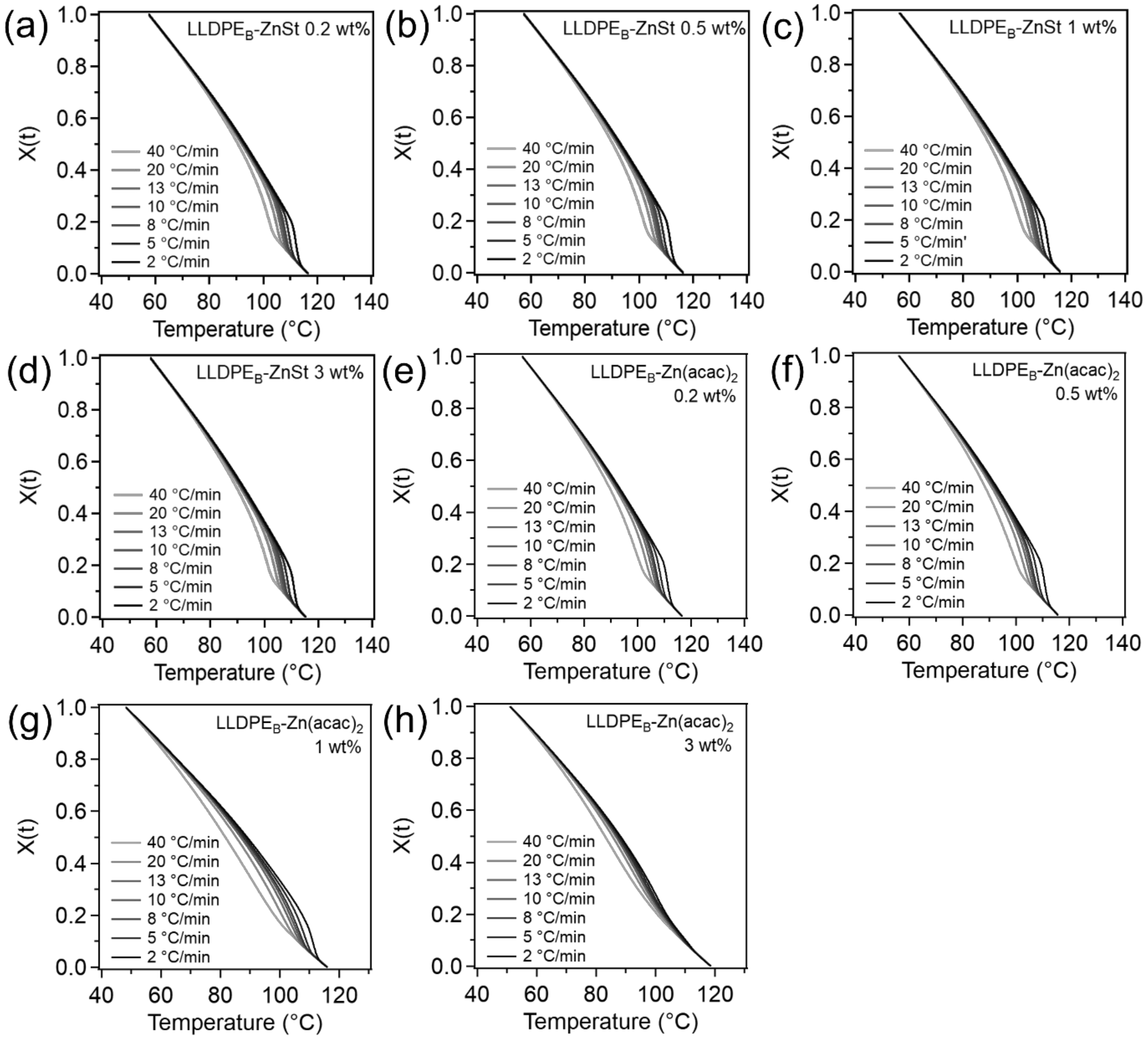

Figure S12. Development of crystallinity with varied cooling rates for (a-d) LLDPE$_B$-ZnSt, and (e-h) LLDPE$_B$-Zn(acac)$_2$ with various catalyst loadings.

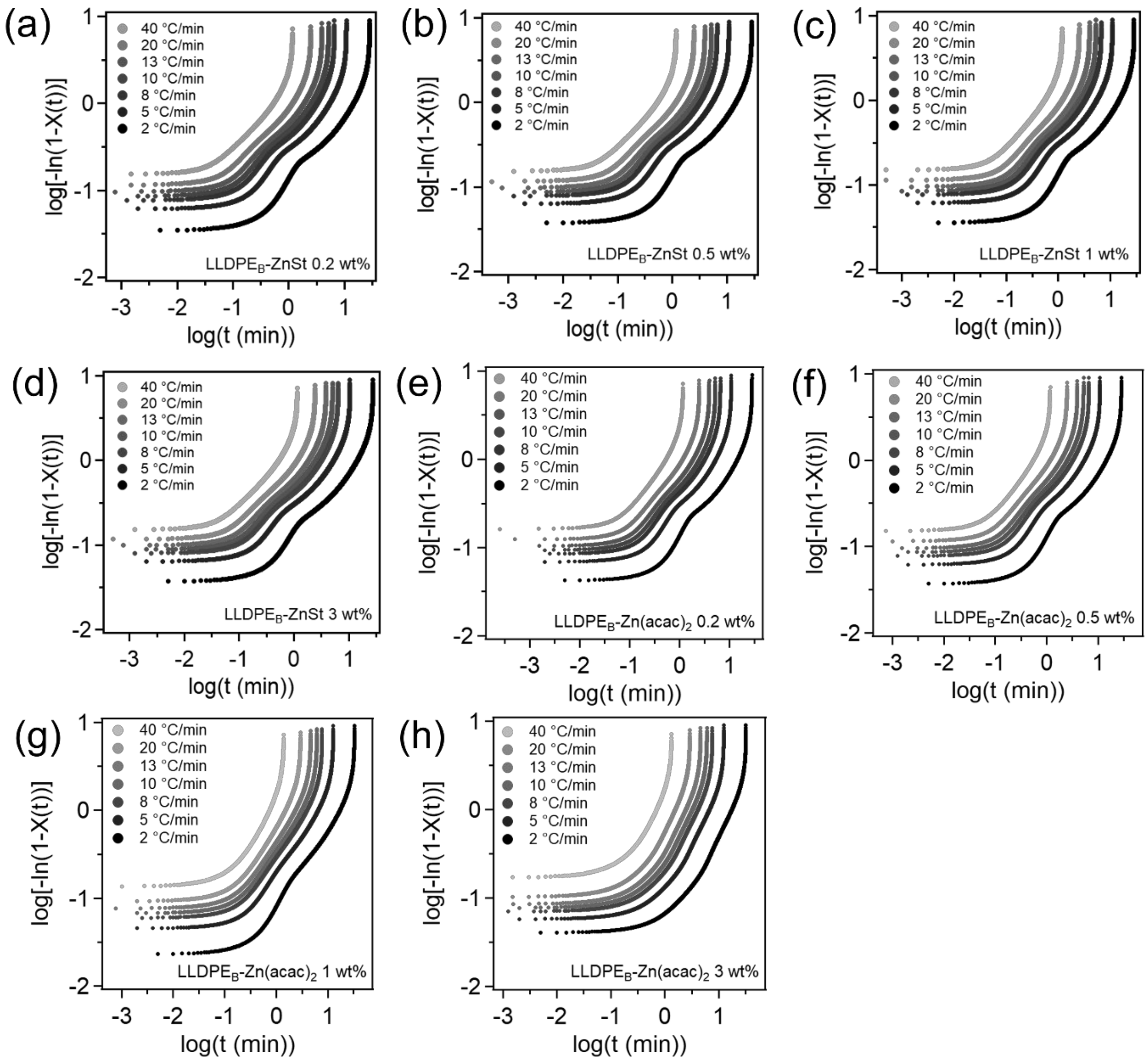

Figure S13. Avrami plots with various cooling rates for (a-d) LLDPE$_B$-ZnSt, and (e-h) LLDPE$_B$-Zn(acac)$_2$ with various catalyst loadings.

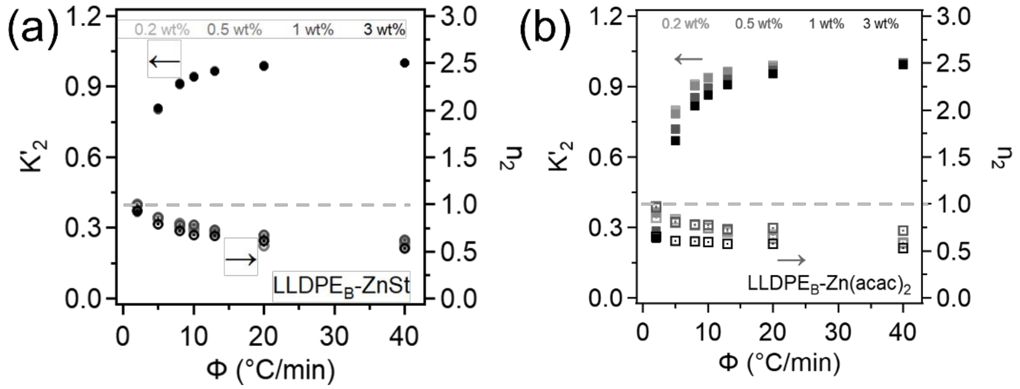

Figure S14. Plots of K'_2 and n_2 with varied cooling rates for (a) $LLDPE_B$-ZnSt and (b) $LLDPE_B$-$Zn(acac)_2$ with various catalyst loadings.

Table S3. Table listing T_m, χ_c, and L for every sample, using the 2nd heat cycle after cooling at 2 °C/min.

Sample	T_m (°C)	χ_c (%)	L (nm)	*Gel fraction (%)*
LLDPE	122.6	39.3	9.1	-
$LLDPE_V$-ZnSt 0.2 wt%	112.0, 122.3	32.8	5.9, 9.0	61.8
$LLDPE_V$-ZnSt 0.5 wt%	112.5, 122.9	33.8	6.0, 9.3	63.0
$LLDPE_V$-ZnSt 1 wt%	111.7, 122.7	31.6	5.8, 9.2	72.8
$LLDPE_V$-ZnSt 3 wt%	111.3, 120.6	29.9	5.8, 8.3	70.5
$LLDPE_V$-$Zn(acac)_2$ 0.2 wt%	111.8, 124.1	29.5	5.8, 9.9	67.9
$LLDPE_V$-$Zn(acac)_2$ 0.5 wt%	112.0, 123.6	34.5	5.9, 9.6	72.7
$LLDPE_V$-$Zn(acac)_2$ 1 wt%	110.7, 123.7	30.8	5.6, 9.7	83.4
$LLDPE_V$-$Zn(acac)_2$ 3 wt%	110.4, 123.6	31.5	5.6, 9.6	80.4
$LLDPE_V$-$Zn(ac)_2$ 1 wt%	111.6, 122.3	32.2	5.8, 9.0	74.0
$LLDPE_V$-$Mn(ac)_2$ 1 wt%	111.9, 122.5	33.4	5.9, 9.1	71.4
$LLDPE_B$-ZnSt 0.2 wt%	122.4	40.8	9.0	-
$LLDPE_B$-ZnSt 0.5 wt%	122.3	40.3	9.0	-
$LLDPE_B$-ZnSt 1 wt%	122.0	39.8	8.8	-
$LLDPE_B$-ZnSt 3 wt%	121.4	40.1	8.6	-
$LLDPE_B$-$Zn(acac)_2$ 0.2 wt%	122.3	39.6	9.0	-
$LLDPE_B$-$Zn(acac)_2$ 0.5 wt%	122.4	40.0	9.0	-
$LLDPE_B$-$Zn(acac)_2$ 1 wt%	125.0	38.4	10	-
$LLDPE_B$-$Zn(acac)_2$ 3 wt%	112.7, 121.8	33.0	6.0, 8.8	-
$LLDPE_B$-$Zn(ac)_2$ 1 wt%	122.3	38.8	9.0	-
$LLDPE_B$-$Mn(ac)_2$ 1 wt%	122.5	38.9	9.1	-

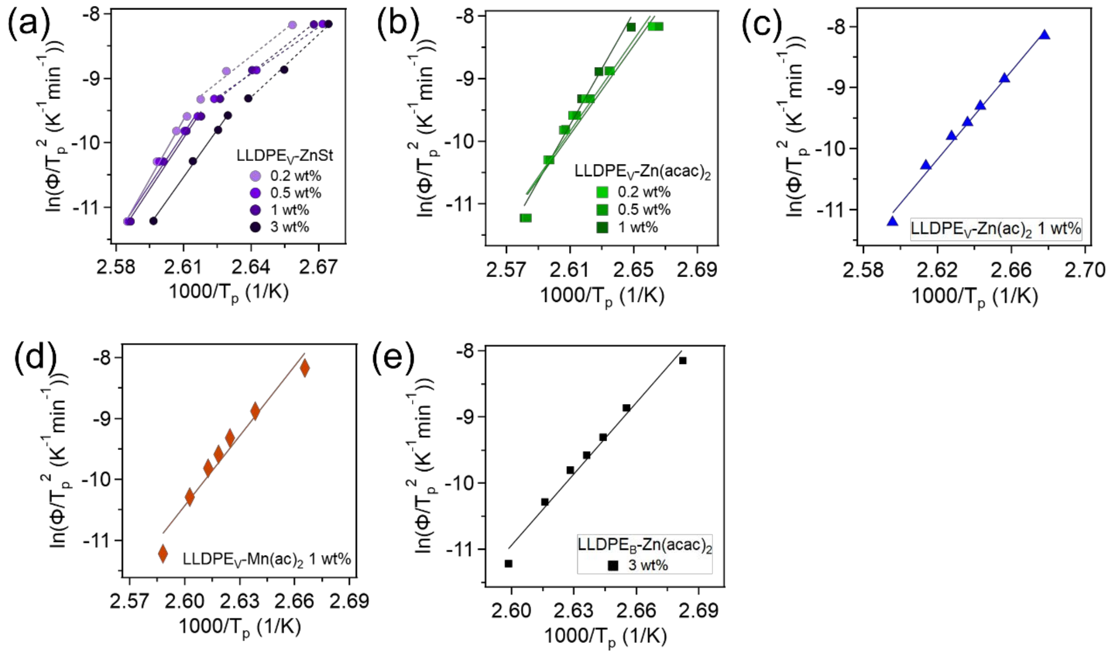

Figure S15. Kissinger plots of $T_{p,1}$ (higher crystallization temperature) for (a) $LLDPE_V$-ZnSt, (b) $LLDPE_V$-Zn(acac)$_2$, (c) $LLDPE_V$-Zn(ac)$_2$, (d) $LLDPE_V$-Mn(ac)$_2$, and (e) $LLDPE_B$-Zn(acac)$_2$.

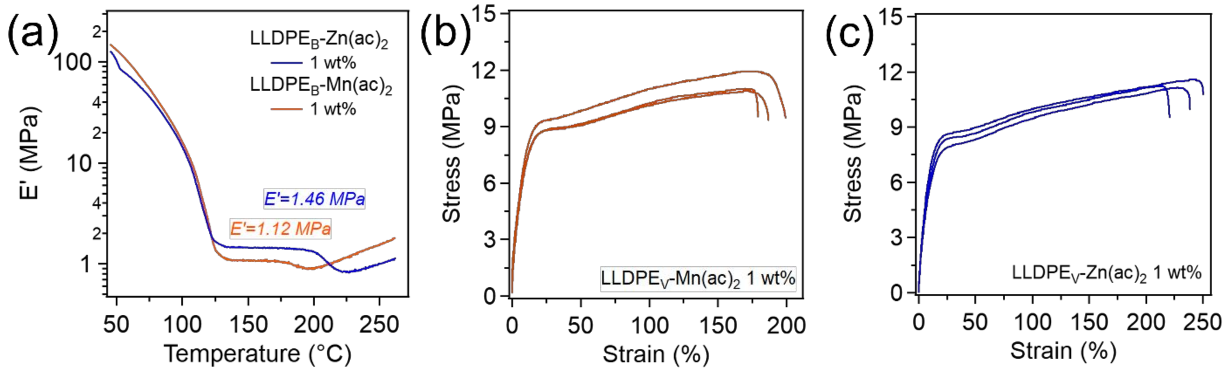

Figure S16. (a) Storage modulus-temperature plot and rubbery modulus and (b,c) stress strain curves for $LLDPE_V$-Zn(ac)$_2$ and $LLDPE_V$-Mn(ac)$_2$ with a catalyst loading of 1 wt%.

Table S4. Table listing tensile properties for $LLDPE_V$-$Zn(ac)_2$ and $LLDPE_V$-$Mn(ac)_2$ with catalyst loadings of 1 wt%, including the average (*AVG*) and standard deviation (*STD*).

Sample	Run	Modulus (MPa)	UTS (MPa)	Strain at break (%)	Toughness (MJ/m^3)
$LLDPE_V$-$Zn(ac)_2$ 1 wt%	1	98.94	11.6	250.4	24.94
	2	89.63	11.3	220.8	21.09
	3	81.16	11.1	238.7	22.41
	AVG	*89.91*	*11.3*	*236.6*	*22.81*
	STD	*7.261*	*0.182*	*12.17*	*1.597*
$LLDPE_V$-$Mn(ac)_2$ 1 wt%	1	136.5	11.0	179.1	17.27
	2	107.2	10.9	186.9	17.90
	3	105.6	12.0	199.4	20.82
	AVG	*116.4*	*11.3*	*188.47*	*18.66*
	STD	*14.20*	*0.484*	*8.361*	*1.547*

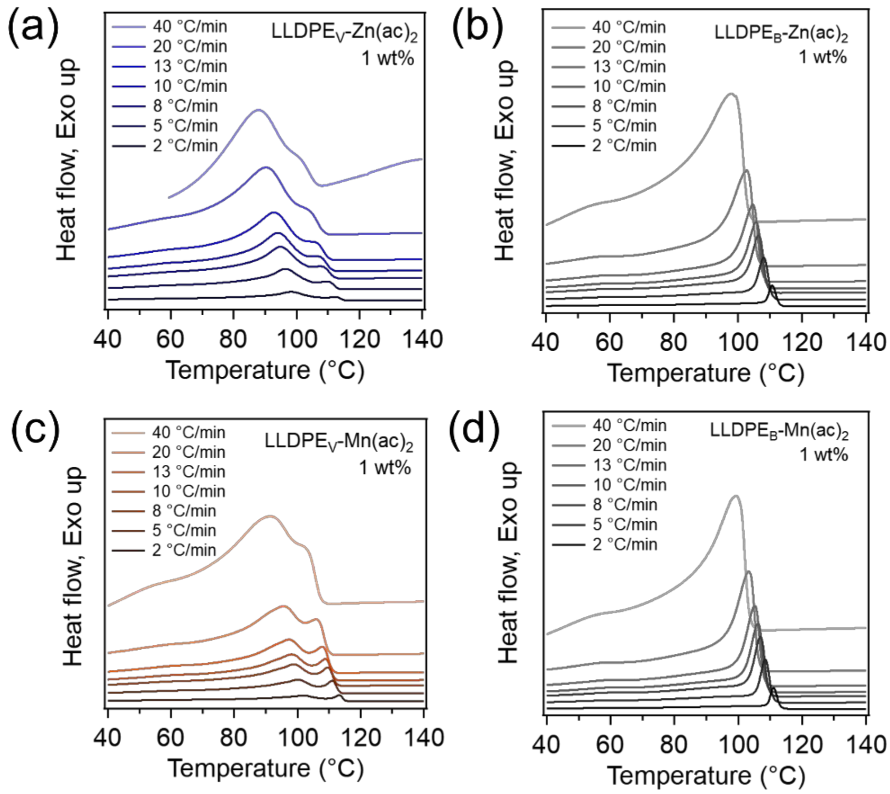

Figure S17. DSC thermograms with varied cooling rates for (a) $LLDPE_V$-$Zn(ac)_2$, (b) $LLDPE_B$-$Zn(ac)_2$, (c) $LLDPE_V$-$Mn(ac)_2$, and (d) $LLDPE_B$-$Mn(ac)_2$, all with a catalyst loading of 1 wt%.

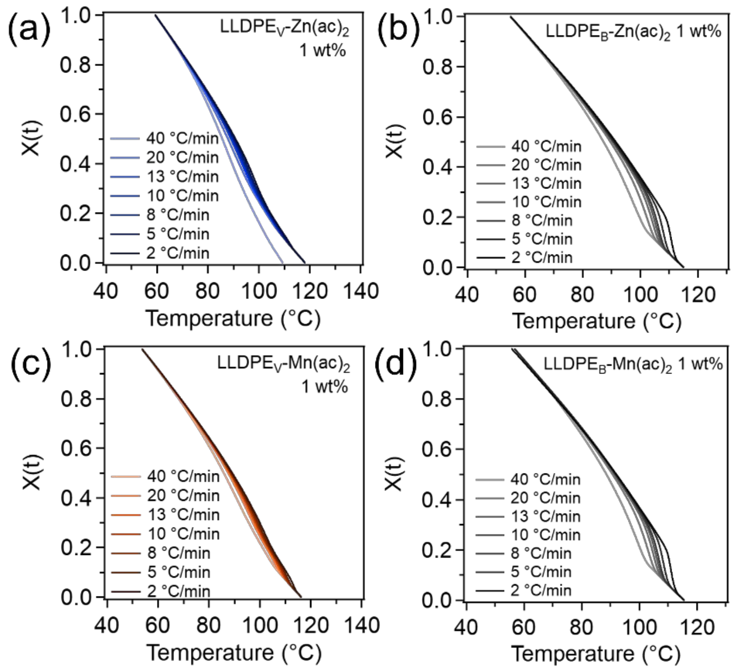

Figure S18. Development of crystallinity with varied cooling rates for (a) LLDPE$_V$-Zn(ac)$_2$, (b) LLDPE$_B$-Zn(ac)$_2$, (c) LLDPE$_V$-Mn(ac)$_2$, and (d) LLDPE$_B$-Mn(ac)$_2$, all with a catalyst loading of 1 wt%.

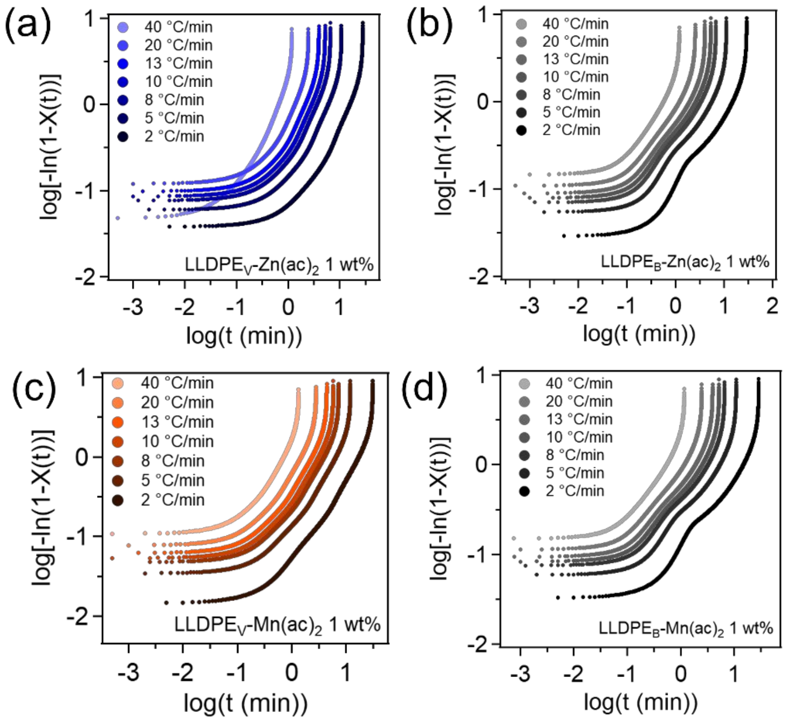

Figure S19. Avrami plots with various cooling rates for (a) LLDPE$_V$-Zn(ac)$_2$, (b) LLDPE$_B$-Zn(ac)$_2$, (c) LLDPE$_V$-Mn(ac)$_2$, and (d) LLDPE$_B$-Mn(ac)$_2$, all with a catalyst loading of 1 wt%.

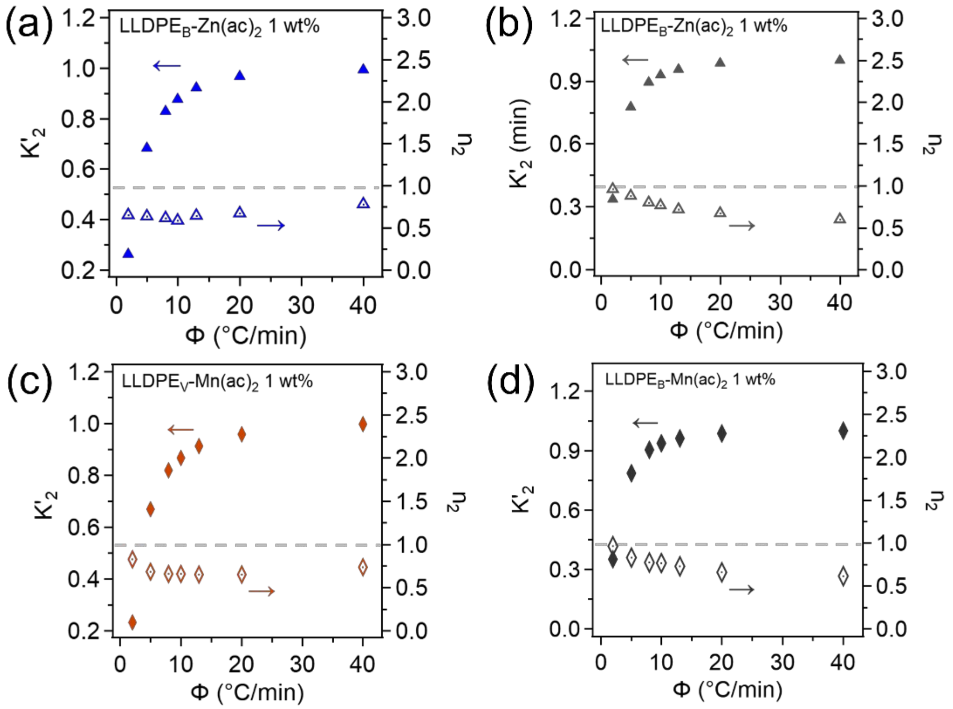

Figure S20. Plots of K'_2 and n_2 with varied cooling rates for (a) LLDPE$_V$-Zn(ac)$_2$, (b) LLDPE$_B$-Zn(ac)$_2$, (c) LLDPE$_V$-Mn(ac)$_2$, and (d) LLDPE$_B$-Mn(ac)$_2$, all with a catalyst loading of 1 wt%.

Table S5. Table listing crystallization activation energies for LLDPE and all vitrimer samples, $T_{p,1}$ is always the higher temperature crystallization peak.

Sample	ΔE (kJ/mol), $T_{p,1}$ 2-40 °C/min	ΔE (kJ/mol), $T_{p,2}$ 2-13 °C/min	ΔE (kJ/mol), $T_{p,2}$ 20-40 °C/min
LLDPE	337.1	-	-
LLDPE$_V$-ZnSt 0.2 wt%	514.8,# 228.6*	557.2	247.5
LLDPE$_V$-ZnSt 0.5 wt%	443.0,# 200.5*	392.6	155.9
LLDPE$_V$-ZnSt 1 wt%	440.2,# 229.2*	561.4	180.1
LLDPE$_V$-ZnSt 3 wt%	406.7,# 269.6*	568.3	211.1
LLDPE$_V$-Zn(acac)$_2$ 0.2 wt%	304.4	530.1	162.5
LLDPE$_V$-Zn(acac)$_2$ 0.5 wt%	295.4	599.6	171.0
LLDPE$_V$-Zn(acac)$_2$ 1 wt%	372.9	794.0	270.5
LLDPE$_V$-Zn(acac)$_2$ 3 wt%	-	767.7	274.5
LLDPE$_V$-Zn(ac)$_2$ 1 wt%	300.8	430.7#	249.8*
LLDPE$_V$-Mn(ac)$_2$ 1 wt%	318.3	526.9	197.1

*#: 2-10 °C/min, *: 13-40 °C/min*

Table S6. Table listing crystallization activation energies for blend samples, $T_{p,1}$ is always the higher temperature crystallization peak.

Sample	Δ*E* (kJ/mol), $T_{p,1}$ 2-13 °C/min	Δ*E* (kJ/mol), $T_{p,1}$ 20-40 °C/min	Δ*E* (kJ/mol), $T_{p,2}$ 2-13 °C/min	Δ*E* (kJ/mol), $T_{p,2}$ 20-40 °C/min
$LLDPE_B$-ZnSt 0.2 wt%	429.8	274.3	-	-
$LLDPE_B$-ZnSt 0.5 wt%	423.3	274.4	-	-
$LLDPE_B$-ZnSt 1 wt%	404.4	235.3	-	-
$LLDPE_B$-ZnSt 3 wt%	424.3	258.8	-	-
$LLDPE_B$-$Zn(acac)_2$ 0.2 wt%	413.1	217.4	-	-
$LLDPE_B$-$Zn(acac)_2$ 0.5 wt%	403.7	191.1	-	-
$LLDPE_B$-$Zn(acac)_2$ 1 wt%	401.2	97.3	-	-
$LLDPE_B$-$Zn(acac)_2$ 3 wt%	298.5*		343.3	120.6
$LLDPE_B$-$Zn(ac)_2$ 1 wt%	378.5	179.5	-	-
$LLDPE_B$-$Mn(ac)_2$ 1 wt%	391.2	200.0	-	-

**: 2-40 ℃/min*

Table S7. Table listing T_{onset}, T_p, $t_{1/2}$, K'_2, and n_2 for all samples with varying cooling rates.

Sample	ϕ (°C/min)	T_{onset} (°C)	T_p (°C)	$t_{1/2}$ (min)	K'_2	n_2
LLDPE	2	113.4	111.4	11.1	0.376	0.960
	5	111.1	108.8	4.53	0.802	0.824
	8	109.7	107.2	2.86	0.913	0.792
	10	109.0	106.4	2.31	0.947	0.778
	13	108.1	105.3	1.79	0.970	0.738
	20	106.4	103.4	1.20	0.996	0.723
	40	104.2	100.8	0.446	1.01	0.892
$LLDPE_V$-ZnSt 0.2 wt%	2	115.4	102.2, 113.7	13.7	0.273	0.669
	5	113.8	100.4, 111.8	5.53	0.693	0.634
	8	112.8	99.40, 110.5	3.50	0.831	0.610
	10	112.3	98.94, 110.0	2.81	0.878	0.609
	13	111.6	98.20, 108.9	2.18	0.918	0.601
	20	110.5	96.88, 107.2	1.45	0.962	0.612
	40	108.2	93.64, 103.0	0.760	0.994	0.625
$LLDPE_V$-ZnSt 0.5 wt%	2	115.6	101.2, 113.6	13.8	0.232	0.798
	5	113.9	98.56, 111.6	5.61	0.668	0.710
	8	112.8	97.12, 110.0	3.55	0.817	0.693
	10	112.3	96.54, 109.1	2.87	0.865	0.639
	13	111.48	95.60, 108.0	2.23	0.908	0.617
	20	110.3	93.86, 105.3	1.49	0.955	0.617
	40	107.7	88.76, 101.1	0.800	0.992	0.640
$LLDPE_V$-ZnSt 1 wt%	2	115.9	98.71, 113.5	14.1	0.219	0.810
	5	114.3	96.97, 111.3	5.72	0.655	0.749
	8	113.3	95.84, 109.8	3.61	0.809	0.695
	10	112.7	95.02, 108.9	2.91	0.861	0.706
	13	111.9	94.00, 107.6	2.27	0.909	0.703
	20	110.5	92.30, 105.6	1.52	0.957	0.684
	40	108.0	87.93, 101.7	0.816	0.992	0.673
$LLDPE_V$-ZnSt 3 wt%	2	114.8	99.40, 112.0	13.9	0.231	0.779
	5	113.2	97.74, 109.4	5.65	0.663	0.666
	8	112.1	96.67, 107.8	3.56	0.816	0.661
	10	111.5	96.11, 107.1	2.87	0.868	0.690
	13	110.8	95.16, 105.8	2.22	0.912	0.670
	20	109.5	93.52, 103.6	1.48	0.958	0.662
	40	107.0	89.78, 100.8	0.787	0.994	0.687
$LLDPE_V$-$Zn(acac)_2$ 0.2 wt%	2	116.3	99.30, 114.1	14.5	0.224	0.750
	5	114.8	97.66, 112.1	5.91	0.648	0.677
	8	113.8	96.52, 110.6	3.74	0.802	0.662
	10	113.3	95.75, 109.7	3.01	0.853	0.629
	13	112.5	94.84, 108.6	2.35	0.898	0.582
	20	111.1	92.96, 106.3	1.58	0.946	0.556
	40	108.3	88.09, 102.6	0.855	0.986	0.539

Sample	ϕ (°C/min)	T_{onset} (°C)	T_p (°C)	$t_{1/2}$ (min)	K'_2	n_2
$LLDPE_V$-$Zn(acac)_2$ 0.5 wt%	2	116.3	99.51, 114.0	14.1	0.222	0.794
	5	114.8	97.98, 111.9	5.74	0.655	0.723
	8	113.7	96.81, 110.4	3.63	0.808	0.676
	10	113.1	96.04, 109.4	2.93	0.860	0.693
	13	112.4	95.21, 108.2	2.28	0.904	0.650
	20	111.0	93.23, 106.5	1.53	0.952	0.615
	40	108.2	88.60, 102.0	0.823	0.987	0.548
$LLDPE_V$-$Zn(acac)_2$ 1 wt%	2	116.8	98.78, 114.3	14.1	0.222	0.789
	5	115.3	97.61, 111.9	5.68	0.655	0.692
	8	114.4	96.75, 110.6	3.58	0.807	0.678
	10	113.9	96.22, 109.8	2.87	0.856	0.602
	13	113.3	95.68, 109.0	2.22	0.900	0.580
	20	112.2	94.46, 107.4	1.47	0.949	0.556
	40	109.8	91.54, 104.4	0.774	0.987	0.547
$LLDPE_V$-$Zn(acac)_2$ 3 wt%	2	117.3	98.33	14.2	0.236	0.741
	5	115.7	97.03	5.73	0.665	0.674
	8	114.9	96.24	3.61	0.811	0.651
	10	114.5	95.74	2.91	0.861	0.635
	13	113.9	95.14	2.25	0.903	0.577
	20	112.7	93.91	1.50	0.952	0.572
	40	110.1	91.04	0.796	0.986	0.490
$LLDPE_V$-$Zn(ac)_2$ 1 wt%	2	114.9	98.31, 112.1	13.7	0.261	0.650
	5	113.2	96.00, 109.4	5.57	0.683	0.636
	8	111.9	94.75, 107.4	3.54	0.827	0.613
	10	111.3	93.95, 106.2	2.87	0.875	0.590
	13	110.4	92.82, 105.2	2.25	0.921	0.647
	20	108.8	90.32, 103.3	1.52	0.967	0.674
	40	106.5	87.75, 100.3	0.595	0.993	0.783
$LLDPE_V$-$Mn(ac)_2$ 1 wt%	2	115.0	101.8, 113.2	13.6	0.232	0.832
	5	113.3	99.74, 111.1	5.49	0.669	0.684
	8	112.2	98.71, 109.6	3.47	0.819	0.656
	10	111.7	98.35, 108.8	2.79	0.868	0.660
	13	111.0	97.56, 107.8	2.16	0.912	0.649
	20	109.7	95.74, 105.8	1.44	0.959	0.654
	40	107.3	91.68, 102.0	0.763	0.997	0.736
$LLDPE_B$-ZnSt 0.2 wt%	2	113.9	112.0	11.7	0.367	0.990
	5	111.6	109.6	4.75	0.803	0.842
	8	110.3	108.2	3.00	0.910	0.755
	10	109.6	107.5	2.41	0.942	0.719
	13	108.8	106.6	1.86	0.970	0.702
	20	107.3	105.1	1.23	0.990	0.635
	40	104.4	102.0	0.651	1.00	0.562

Sample	ϕ (°C/min)	T_{onset} (°C)	T_p (°C)	$t_{1/2}$ (min)	K'_2	n_2
$LLDPE_B$-ZnSt 0.5 wt%	2	113.5	111.5	11.7	0.373	1.00
	5	111.2	109.1	4.73	0.807	0.857
	8	109.9	107.7	2.99	0.915	0.797
	10	109.2	106.9	2.40	0.945	0.750
	13	108.3	106.1	1.86	0.967	0.700
	20	106.8	104.4	1.23	0.987	0.613
	40	104.0	101.4	0.658	1.00	0.562
$LLDPE_B$-ZnSt 1 wt%	2	113.1	111.1	11.7	0.366	0.996
	5	110.8	108.7	4.76	0.801	0.857
	8	109.5	107.1	3.01	0.909	0.778
	20	108.8	106.4	2.42	0.944	0.783
	13	108.0	105.4	1.87	0.967	0.728
	20	106.4	103.7	1.25	0.990	0.677
	40	103.7	100.2	0.667	1.00	0.626
$LLDPE_B$-ZnSt 3 wt%	2	112.6	111.0	11.5	0.381	0.929
	5	110.4	108.7	4.67	0.808	0.790
	8	109.0	107.2	2.95	0.912	0.718
	10	108.3	106.5	2.37	0.940	0.679
	13	107.5	105.6	1.84	0.967	0.667
	20	106.1	104.1	1.22	0.988	0.613
	40	103.5	100.9	0.643	1.00	0.531
$LLDPE_B$-$Zn(acac)_2$ 0.2 wt%	2	113.2	111.2	11.9	0.366	0.851
	5	110.9	108.6	4.84	0.799	0.834
	8	109.5	107.1	3.06	0.910	0.782
	10	108.8	106.4	2.46	0.940	0.743
	13	107.9	105.3	1.91	0.964	0.699
	20	106.4	103.4	1.27	0.990	0.671
	40	103.4	99.59	0.689	1.00	0.583
$LLDPE_B$-$Zn(acac)_2$ 0.5 wt%	2	112.7	110.7	11.7	0.361	0.978
	5	110.5	108.1	4.76	0.784	0.803
	8	109.1	106.6	3.02	0.902	0.774
	10	108.4	105.8	2.43	0.935	0.745
	13	107.5	104.7	1.89	0.961	0.716
	20	105.9	103.1	1.26	0.983	0.631
	40	103.1	98.71	0.692	1.00	0.587
$LLDPE_B$-$Zn(acac)_2$ 1 wt%	2	113.6	111.3	13.5	0.285	0.964
	5	111.5	108.4	5.53	0.719	0.811
	8	110.1	106.5	3.53	0.853	0.779
	10	109.4	105.2	2.68	0.896	0.773
	13	108.5	103.6	2.25	0.930	0.729
	20	107.1	99.90	1.54	0.969	0.743
	40	103.7	91.30	0.861	0.997	0.715

Sample	ϕ (°C/min)	T_{onset} (°C)	T_p (°C)	$t_{1/2}$ (min)	K'_2	n_2
$LLDPE_B$-$Zn(acac)_2$ 3 wt%	2	114.7	100.5, 111.7	15.2	0.253	0.655
	5	113.1	98.00, 109.1	6.18	0.671	0.611
	8	112.0	96.38, 107.3	3.92	0.816	0.604
	10	111.5	95.38, 106.2	3.16	0.864	0.597
	13	110.8	94.09, 105.1	2.47	0.907	0.578
	20	109.3	91.79, 103.5	1.66	0.954	0.575
	40	104.1	85.21, 99.66	0.893	0.992	0.992
$LLDPE_B$-$Zn(ac)_2$ 1 wt%	2	112.8	110.7	11.8	0.335	0.960
	5	110.5	108.1	4.81	0.777	0.876
	8	109.1	106.5	3.04	0.894	0.799
	10	108.4	105.7	2.45	0.929	0.763
	13	107.5	104.6	1.91	0.956	0.716
	20	105.9	102.7	1.28	0.984	0.672
	40	103.0	98.07	0.697	1.00	0.597
$LLDPE_B$-$Mn(ac)_2$ 1 wt%	2	113.1	111.0	11.8	0.352	0.963
	5	110.7	108.5	4.71	0.787	0.831
	8	109.4	106.9	2.98	0.903	0.777
	10	108.7	106.2	2.40	0.938	0.771
	13	107.8	105.1	1.86	0.963	0.728
	20	106.2	103.3	1.24	0.986	0.660
	40	103.4	99.31	0.676	1.00	0.616
$LLDPE_V$-insol	2	105.0	96.73	-	-	-
	5	104.3	94.88	-	-	-
	10	103.7	92.74	-	-	-
	20	102.0	89.59	-	-	-
	40	98.75	84.76	-	-	-
$LLDPE_V$-insol 1 wt% ZnSt	2	101.6	97.21	-	-	-
	5	101.1	95.45	-	-	-
	10	100.1	93.42	-	-	-
	20	99.26	90.71	-	-	-
	40	97.70	86.15	-	-	-
